# Hydrogels as Soft Ionic Conductors in Flexible and Wearable Triboelectric Nanogenerators

**DOI:** 10.1002/advs.202106008

**Published:** 2022-02-20

**Authors:** Yinghong Wu, Yang Luo, Tyler J. Cuthbert, Alexander V. Shokurov, Paul K. Chu, Shien‐Ping Feng, Carlo Menon

**Affiliations:** ^1^ Biomedical and Mobile Health Technology Lab Department of Health Sciences and Technology ETH Zurich Zurich 8008 Switzerland; ^2^ Department of Physics Department of Materials Science and Engineering and Department of Biomedical Engineering City University of Hong Kong Hong Kong 999077 China; ^3^ Department of Mechanical Engineering The University of Hong Kong Hong Kong 999077 China; ^4^ Department of Advanced Design and Systems Engineering City University of Hong Kong Kowloon Hong Kong 999077 China

**Keywords:** flexible and wearable applications, hydrogel, ionic conductivity, current collector, triboelectric nanogenerator

## Abstract

Flexible triboelectric nanogenerators (TENGs) have attracted increasing interest since their advent in 2012. In comparison with other flexible electrodes, hydrogels possess transparency, stretchability, biocompatibility, and tunable ionic conductivity, which together provide great potential as current collectors in TENGs for wearable applications. The development of hydrogel‐based TENGs (H‐TENGs) is currently a burgeoning field but research efforts have lagged behind those of other common flexible TENGs. In order to spur research and development of this important area, a comprehensive review that summarizes recent advances and challenges of H‐TENGs will be very useful to researchers and engineers in this emerging field. Herein, the advantages and types of hydrogels as soft ionic conductors in TENGs are presented, followed by detailed descriptions of the advanced functions, enhanced output performance, as well as flexible and wearable applications of H‐TENGs. Finally, the challenges and prospects of H‐TENGs are discussed.

## Graphical abstract

Owing to its stretchability, biocompatibility, and tunable ionic conductivity, hydrogels have gained increasing attention as current collectors in triboelectric nanogenerators (TENGs). However, a comprehensive review concerning the development of hydrogel‐based TENGs (H‐TENGs) is still lacking. This paper summarizes the recent progress and prospect of H‐TENGs for flexible and wearable applications. Challenges and opportunities of this exciting area are highlighted.

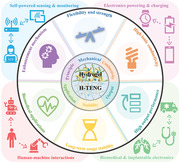

## 1 Introduction

Nowadays, the growing popularity of next‐generation portable and wearable electronics has spurred increasing demands for power sources.^[^ ^1^, ^2^, ^3^, ^4^, ^5^ ^]^ However, the rigid/complicated configuration, large size, and environmentally unfriendly properties of most traditional power supplies no longer satisfy the critical requirements of wearable electronics. Therefore, researchers have made numerous efforts to develop soft and green power sources for wearable applications in recent years.^[^ ^6^, ^7^, ^8^, ^9^ ^]^ Among them, flexible triboelectric nanogenerators (TENGs) have aroused interest because of their simple structure and fabrication, light weight, high output, and low cost.^[^ ^10^, ^11^, ^12^, ^13^, ^14^, ^15^ ^]^

In addition to triboelectric materials, electrodes are also important to the integration of TENGs in flexible and wearable applications. Typically, there are four types of electrodes in flexible TENGs: i) metal sheets; ii) carbon sheets; iii) conductive polymer films; and iv) hydrogel films.^[^ ^16^, ^17^, ^18^, ^19^, ^20^ ^]^ In spite of the high conductivity, metal sheets are usually not suitable for flexible and wearable applications because of the limited flexibility and stretchability. Similar drawbacks also plague carbon sheets that show lower conductivity despite a lower cost. Although conductive polymer films can be stretched if they are fabricated on flexible substrates, the synthesis is usually complicated and the conductivity is poor. In contrast, hydrogels possess unique advantages such as high transparency, stretchability, biocompatibility, as well as small environmental impact.^[^ ^21^, ^22^, ^23^, ^24^ ^]^ More importantly, compared to the electronic conductivity exhibited by the metal sheets/carbon sheets/conductive polymer films, hydrogels have ionic conductivity. This property allows fine‐tuning and optimization of the resistance and charge‐carrier density, selection of chemical ionic species utilized in the material, as well as integration of biological and electronic systems.^[^ ^25^, ^26^ ^]^ Consequently, hydrogels have possessed a large potential to improve the performance and integration of soft electrodes/conductors in TENGs for wearable and biomedical electronics.

In 2017, Xu et al.^[^ ^27^ ^]^ reported the first hydrogel‐based TENG (H‐TENG) consisting of a polyvinyl alcohol (PVA) hydrogel as the conductor and polydimethylsiloxane (PDMS) as the triboelectric layer. The PVA‐based H‐TENG worked as a self‐powered human motion sensor because it can harvest biomechanical energy from stretching, bending, and twisting. In the same year, Pu et al.^[^ ^28^ ^]^ reported the first ionic hydrogel (polyacrylamide (PAM)/LiCl) that possessed high stretchability and transparency as the current collector. The skin‐like PAM/LiCl H‐TENG boosting human motion energy harvesting and touch sensing has great potential in artificial skins, wearable electronics, and soft robots. Since then, H‐TENGs have garnered more attention leading to the rapid development of the output performance and long‐term stability, which benefits the extended applications.

**Figure 1:**
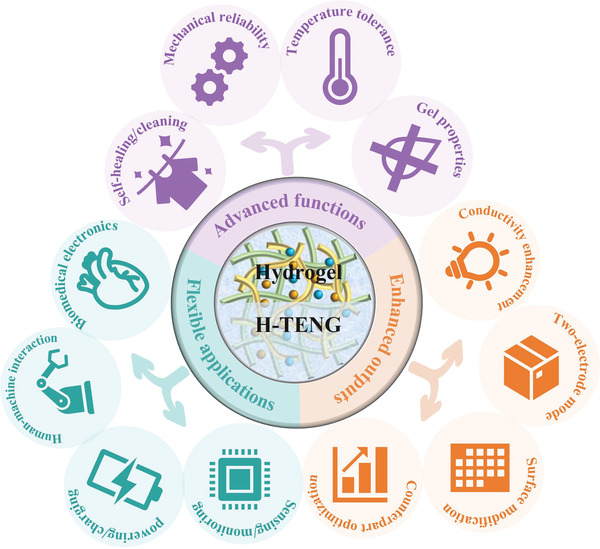
Development of H‐TENGs: advanced functions, enhanced outputs, and flexible and wearable applications.

Up until now, there have been several reviews highlighting the development of hydrogels as soft conductors, but they mainly focus on the properties and applications to sensing devices such as biosensors, force/strain sensors, and gas sensors.^[^ ^26^, ^29^, ^30^, ^31^, ^32^, ^33^ ^]^ A comprehensive review focusing on hydrogels as ionic conductors in flexible TENGs with a detailed discussion on the achievements and challenges is still lacking. In this review, we summarize the recent progress and current status of hydrogels as current collectors in TENGs. Specifically, the advantages of using hydrogels as ionic conductors are discussed. By classifying the types of hydrogels in flexible TENGs with emphasis on conductive additives, the advanced functions and outputs (**Figure** **1**) to improve the long‐term stability of H‐TENGs are highlighted. Last but not least, wearable applications of H‐TENGs are presented, and finally, the challenges and opportunities for future research and development of H‐TENGs are discussed.

## 2 Fundamentals of TENGs

Based on the mechanisms of contact electrification and electrostatic induction, TENGs convert ambient mechanical energy into valuable electricity.^[^ ^10^ ^]^ Regardless of the type (metal/semiconductor/insulator) and state (solid/liquid/gas) of the triboelectric materials, it is gradually accepted that electron transfer plays a dominant role in contact electrification.^[^ ^34^, ^35^, ^36^ ^]^

**Figure 2:**
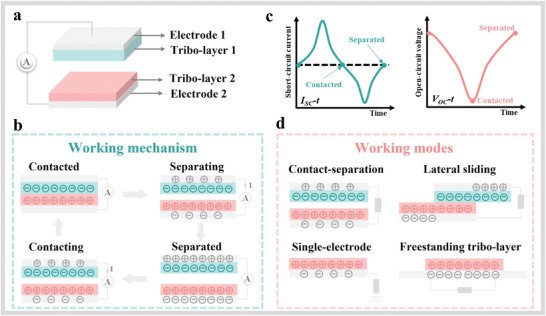
Fundamentals of TENGs: a) schematic diagram of TENGs based on contact‐separation mode, b) working mechanism of contact‐separation TENGs, c) *I*_SC_–*t* and *V*_OC_–*t* curves, and d) working modes of TENGs.

**Figure** **2**a depicts a typical contact‐separation TENG consisting of two different triboelectric layers (tribo‐layers) and two attached electrodes. When the two tribo‐layers come into contact under an external force (Figure 2b), they are oppositely charged based on the contact electrification and there is no potential difference at this stage. When the force is released, these two layers separate from each other. The static charges remaining on the surface produce the potential difference between the two electrodes, causing the flowing of free charges to generate a current signal. When the layers are fully separated, a new balance is achieved between each layer and the attached electrode. As soon as the force is applied again, the charges among the electrodes flow back quickly and an opposite current signal is created. Therefore, alternating current and voltage signals as schematically shown in Figure 2c are obtained during the contact and separation cycles.

In addition to the vertical contact‐separation mode with stable and high output, Figure 2d also shows three other working modes commonly used in TENGs, including the lateral sliding mode, freestanding tribo‐layer mode, and single‐electrode mode. Based on the same configuration but different friction direction, electricity can be generated by lateral sliding of the two tribo‐layers. In long‐term usage, a potential issue of wear and tear needs to be considered. Given the advantages of high conversion efficiency and movable working environments, the freestanding tribo‐layer mode is often used in TENGs with the rotary and complex configurations for wider applications.^[^ ^37^, ^38^ ^]^ Composed of a tribo‐layer and ground electrode, the single‐electrode mode requires simpler structure/fabrication and can work freely in various occasions. Therefore, most reported H‐TENGs were based on the single‐electrode mode for flexible and wearable applications.

## 3 Advantages of Hydrogels as Ionic Conductors in TENGs

As current collectors in TENGs, hydrogels have many advantages which are highlighted in this section. The reasons why they provide additional value to TENGs are also discussed.

### 3.1 High Transparency

**Figure 3:**
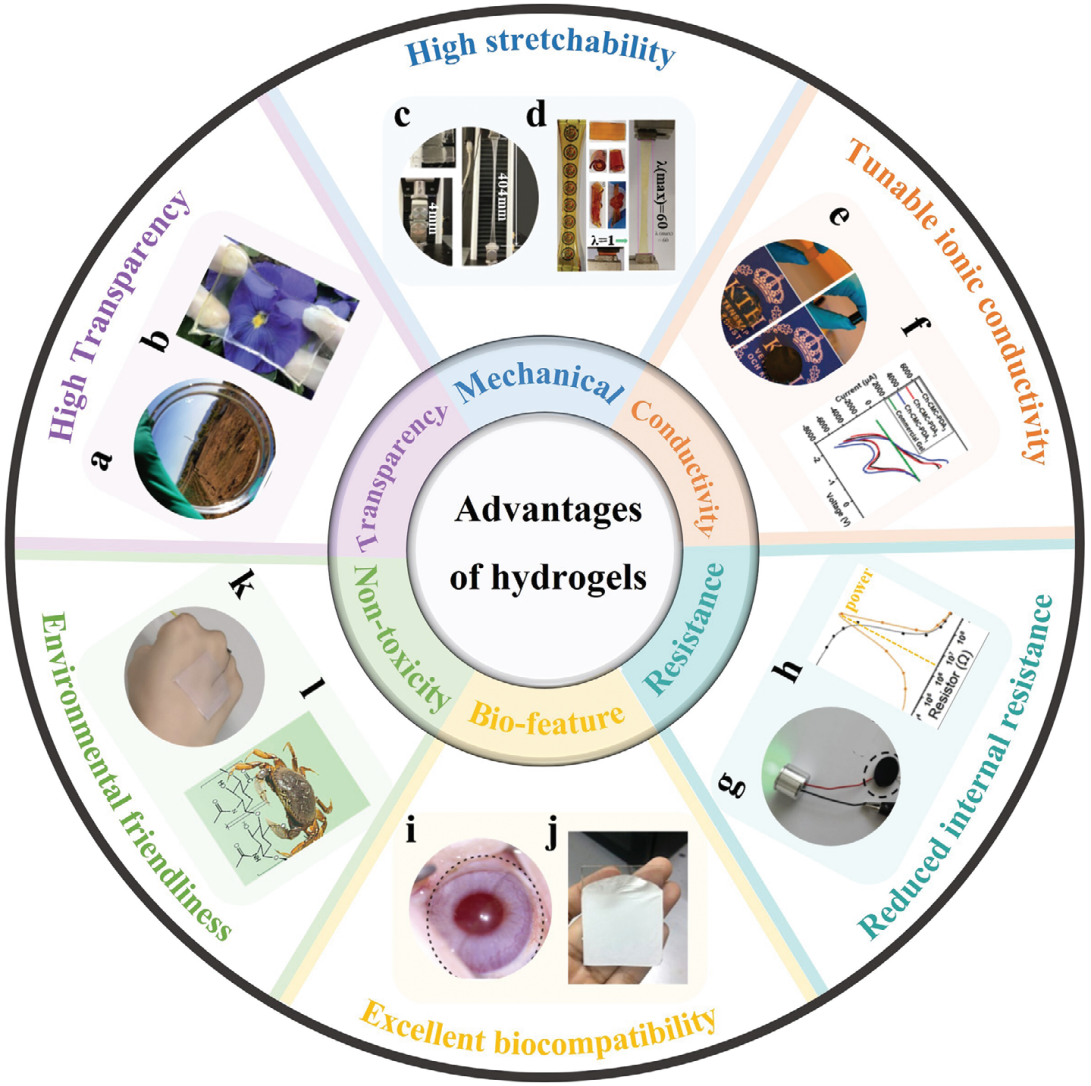
Advantages of hydrogels as ionic conductors in TENGs: a) optical image of the PAM/cyclodextrin hydrogel. Reproduced with permission.^[^ ^42^ ^]^ Copyright 2021, Elsevier. b) Photograph of the transparent cellulose/PVA hydrogel. Reproduced with permission.^[^ ^43^ ^]^ Copyright 2020, Royal Society of Chemistry. c) Hydrogel specimen for tensile testing. Reproduced with permission.^[^ ^44^ ^]^ Copyright 2016, Wiley‐VCH. d) Optical images of the PAM/PDA hydrogel with flexible and stretchable features. Reproduced with permission.^[^ ^49^ ^]^ Copyright 2020, Royal Society of Chemistry. e) Photograph of the AcGGM/90C hydrogel with/without AP. Reproduced with permission.^[^ ^50^ ^]^ Copyright 2014, American Chemical Society. f) Current–voltage characteristics of Ch–CMC–PDAx and commercial gel. Reproduced with permission.^[^ ^51^ ^]^ Copyright 2021, Royal Society of Chemistry. g) Circuit comprising PMZn‐GL hydrogel with a green LED indicator. Reproduced with permission.^[^ ^53^ ^]^ Copyright 2021, Wiley‐VCH. h) Output power of the TENG/Ecoflex device versus resistive load. Reproduced with permission.^[^ ^54^ ^]^ Copyright 2019, Elsevier. i) Photograph of the albino rabbit eye wearing the Au_c_‐NM/HyCL(PEDOT) device. Reproduced with permission.^[^ ^70^ ^]^ Copyright 2019, American Chemical Society. j) Photograph of the BC/ZnO nanocomposite hydrogel treated with amino‐silane. Reproduced with permission.^[^ ^71^ ^]^ Copyright 2020, American Chemical Society. k) Photograph of the AVG/NaCl‐based device. Reproduced with permission.^[^ ^73^ ^]^ Copyright 2020, Elsevier. l) Molecular structure of nanochitin as a biopolymer. Reproduced with permission.^[^ ^64^ ^]^ Copyright 2021, American Chemical Society.

The transparency of hydrogels is an important characteristic in optical and wearable applications that require visual control such as screens, user‐readable sensors, etc.^[^ ^39^, ^40^ ^]^ To achieve high transparency, the water content of the pre‐polymerization mixture should not exceed the maximum water uptake of the hydrogel.^[^ ^41^ ^]^ If it is exceeded, the resulting hydrogel will phase separate and become opaque because of the scattering of light by these polymer‐rich phases (often referred to as pores). Transparency is also an important parameter affecting visual transmission of information and esthetics in wearable and biomedical applications of TENGs. For example, if the H‐TENG is implanted near the heart, good transparency renders it possible for doctors to obtain detailed information for diagnosis. When considering the H‐TENG as electronic skin in people, the transparent one is preferred for cosmetic reasons. Therefore, many reported works have emphasized the high transparency of H‐TENGs, such as PAM/cyclodextrin hydrogel (**Figure** **3**a) prepared by Jiang et al.^[^ ^42^ ^]^ and cellulose/PVA hydrogel (Figure 3b) developed by Wang et al.,^[^ ^43^ ^]^ albeit sometimes without neither requirement nor justification. With that in mind, transparency may allow seamless integration with other devices that can leverage both energy harvesting and optical information acquisition by the H‐TENG. Some researchers have indicated the specific advantages of transparent energy harvesters. For example, Pu et al.^[^ ^28^ ^]^ have developed integrated H‐TENGs and touch sensing without optical information loss. Leveraging transparency should be a focus of future research; exploitation of this unique characteristic may foster integration of H‐TENG energy harvesters into a larger section of everyday devices.

### 3.2 High Stretchability

Another important characteristic of hydrogels is the stretchability. Some hydrogels can be strained over 1000%, which is imparted through the chemical composition of the polymer matrix and swelling of the network with water. As shown in Figure 3c, a PAM hydrogel was synthesized by cross‐linking with a hydrophobic acrylate, which could undergo stretching from 4 to 404 mm without fracturing.^[^ ^44^ ^]^ In wearable sensing and monitoring applications, good flexibility and stretchability are important factors when selecting materials composition. Particularly, flexible TENGs are often utilized as angle sensors to monitor elbow or knee bending.^[^ ^45^, ^46^, ^47^, ^48^ ^]^ Compared to bare hydrogel sensors, the H‐TENG strain sensors can not only power electronics based on the voltage signals, protect the inside hydrogel from being contaminated/damaged, but also retard its water loss and further be utilized in wearable applications. The high flexibility and stretchability of hydrogels also provide suitability as electrodes in TENGs for motion energy harvesting requiring high strain values, which are unique advantages that other flexible electrodes can not achieve. For example, the PAM/polydopamine(PDA) hydrogel developed by Long et al.^[^ ^49^ ^]^ shown in Figure 3d was not damaged when rolled, twisted, and knotted. In fact, this PAM/PDA hydrogel can be stretched to 6000% of the original length and is regarded to have “super stretchability”. Therefore, even after substantial stretching, the PAM/PDA H‐TENG can maintain stable and excellent output.

### 3.3 Tunable Ionic Conductivity

In comparison with other flexible electrodes with high conductivity in TENGs, one of the unique advantages of hydrogels is the tunable ionic conductivity. It is convenient to introduce conductive additives—such as salts and carbon‐based materials—into the hydrogel precursor to tailor the conductivity. For instance, addition of 40% of aniline pentamer into acetyl‐galactoglucomannan hydrogel changeed the appearance from transparent to black (Figure 3e), and the conductivity increased from “undetectable” to 1.58 µs cm^−1^.^[^ ^50^ ^]^ As shown in Figure 3f, addition of PDA in chitosan/cellulose (Ch‐CMC) also enhanced the conductivity gradually with increasing quantity.^[^ ^51^ ^]^ When considering hydrogels as the electrodes in TENGs for bioelectronic applications, the obtained electro‐cardiogram signal of the Ch‐CMC‐PDA H‐TENG was much higher than that of the commercial gel‐based TENG. Moreover, even after the hydrogel synthesis, it is still possible to improve the conductivity by a simple immersion process. In this case, the hydrogel is soaked in a salt solution to cause ion transfer into the hydrogel. For example, Gao et al.^[^ ^52^ ^]^ have immersed the PVA hydrogel into a Fe_2_(SO_4_)_3_ solution; the resulting multi‐crosslinked hydrogel showed a good conductivity of 0.6 S m^−1^ at 25 ℃ and served as a flexible strain and pressure sensor for human motions monitoring.

### 3.4 Reduced Internal Resistance

Although the conductivity of hydrogels is lower than that of metal electrodes, the internal resistance is still acceptable. For example, when connecting the PVA/MXene hydrogel prepared by Feng et al.^[^ ^53^ ^]^ into a closed circuit, the green LED could be lit up (Figure 3g). Notably, it is found that the internal resistance of many H‐TENGs is lower than that of common metal electrode‐based TENGs, which may benefit the power circuit management and powering/charging of portable/wearable electronics. As shown in Figure 3h, the highest output power of Ag nanowires (Ag NWs)/PVA H‐TENG was obtained at an external resistance of 3.3 MΩ.^[^ ^54^ ^]^ This indicates the matched internal resistance of the device was only 3.3 MΩ, which is tens or hundreds times less than that of common reported TENGs.^[^ ^55^, ^56^, ^57^, ^58^ ^]^ Similar results have been observed from other H‐TENGs as well,^[^ ^59^, ^60^, ^61^, ^62^, ^63^, ^64^ ^]^ with intriguing applications such as wireless real‐time communication, large capacitors charging, temperature sensing, as well as human motion monitoring. The stronger mechanical adhesion and better reliability between the triboelectric layer and hydrogel resulted in the better charge induction. Many hydrogels in H‐TENGs attach strongly to the triboelectric layer by forming chemical bonds via the benzophenone (BP) treatment. Even in the absence of a chemical treatment, the flexibility and large water content of hydrogels enable consistent contact to the triboelectric layers to promote electrostatic induction and charge transfer.

### 3.5 Excellent Biocompatibility

Biocompatibility is an essential requirement for TENGs in biomedical and implantable applications. With this in mind, most reported hydrogels have excellent biocompatibility making them suitable for tissue regeneration, artificial skins, health monitoring, and drug delivery.^[^ ^65^, ^66^, ^67^, ^68^, ^69^ ^]^ For example, Wei et al.^[^ ^70^ ^]^ have fabricated metal nanofiber mesh‐based hydrogel contact lenses with excellent gas permeability, wettability, and transparency. As shown in Figure 3i, after inserting the hydrogel device in an albino rabbit eye for 12 h, no obvious corneal abrasion or irritation was observed. The good biocompatibility is also valuable to wearable and biomechanical applications of H‐TENGs. Figure 3j showed a biocompatible dried hydrogel film composed of bacterial cellulose and ZnO; the fabricated bio‐TENG had excellent bioinspired and biodegradable properties.^[^ ^71^ ^]^ Moreover, the compatibility makes it possible for the ionic conductive hydrogels to be used in bioelectronics, sensors, as well as mechanical actuators.^[^ ^72^ ^]^

### 3.6 Environmental Friendliness

Unlike other properties, environmental friendliness is a parameter related to the impact of the current/future materials we develop and implement into next‐generation technologies. Therefore, research of H‐TENGs also should consider the impact of the raw material source, chemicals used in fabrication, end of life, as well as recycling technology. For example, Wu et al.^[^ ^73^ ^]^ have developed a sustainable TENG consisting of biocompatible and renewable aloe vera gel and silicone rubber (Figure 3k), which could be safely worn on skin to detect body motions and monitor facial expressions. Some researchers have attempted to use natural materials to prepare hydrogels for TENGs. As one of the most abundant biopolymers, chitin is the main structural component in living organisms, for instance, carb as seen in Figure 3l. Interestingly, a polyacrylic acid (PAA)/nanochitin hydrogel was synthesized via a facial and green approach by Jing et al.^[^ ^64^ ^]^ The developed chitin‐based sensors and TENGs can be used to monitor throat movement during speaking, body joint bending, as well as finger gestures. This provides an alternative raw material presenting a direction that can move away from petroleum‐based raw materials.

## 4 Types of Hydrogels as Ionic Conductors in TENGs

**Figure 4:**
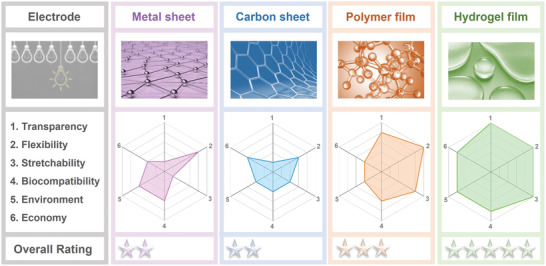
Comparison of various flexible electrodes in TENGs for flexible and wearable applications: metal sheet, carbon sheet, conductive polymer film, and hydrogel film.

Generally speaking, there is no special requirements for electrodes in common TENGs, expect for the conductivity and attachment to tribo‐layers. However, in flexible and wearable applications, the electrodes in TENGs need to meet several requirements: i) tunable conductivity to obtain acceptable output to detect human motions and power portable electronics; ii) flexibility and stretchability to harvest bending/twisting energy while offering wearing comfort; iii) non‐toxicity to ensure the health and safety of the users; and iv) transparency required for visual transmission of information and aesthetics. In this regard, hydrogel is the ideal choice for flexible TENGs, compared to other flexible electrodes such as metal sheets, carbon sheets, and conductive polymer films, as shown in **Figure** **4**. Typically, hydrogels as electrode materials for flexible TENGs can be divided into the following categories: pure hydrogels, salt‐modified hydrogels, carbon‐modified hydrogels, and other hydrogels.

### 4.1 Pure Hydrogels

Pure hydrogels in this review are defined as hydrogels that only contain polymers but no additives such as salts and carbon materials. Although pure hydrogels are normally transparent, flexible, and non‐toxic, their conductivities are relatively low and non‐tunable. Based on the network structure, pure hydrogels can be divided into single network and double network ones. The most popular single network hydrogels for TENGs are PAM and PVA. PVA hydrogels were first reported for versatile energy harvesting based on triboelectric effect,^[^ ^27^ ^]^ and Mi et al.^[^ ^74^ ^]^ have employed the PAM hydrogel as the current collector in two‐electrode TENGs.

**Figure 5:**
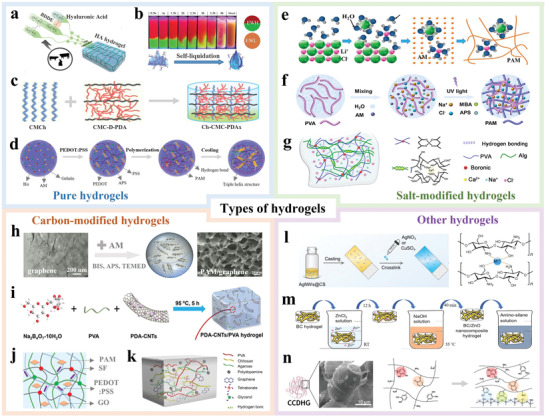
Types of hydrogels as ionic conductors in TENGs: a) schematic diagram showing fabrication of the HA hydrogel. Reproduced with permission.^[^ ^75^ ^]^ Copyright 2021, Wiley‐VCH. b) Photographs and schematic illustration of the self‐liquidation process of gelation. Reproduced with permission.^[^ ^76^ ^]^ Copyright 2020, Wiley‐VCH. c) Schematic showing the preparation of the Ch‐CMC‐PDA hydrogel. Reproduced with permission.^[^ ^51^ ^]^ Copyright 2021, Royal Society of Chemistry. d) Schematic illustration of the synthetic procedures of MGP CHs. Reproduced with permission.^[^ ^80^ ^]^ Copyright 2020, Elsevier. e) Schematic illustration of PAM/LiCl hydrogel synthesis. Reproduced with permission.^[^ ^81^ ^]^ Copyright 2021, Elsevier. f) Schematic illustration of the synthesis process of PAM/PVA/NaCl hydrogels. Reproduced with permission.^[^ ^63^ ^]^ Copyright 2021, American Chemical Society. g) Schematic illustration of the synthesis of the PVA/SA hydrogel. Reproduced with permission.^[^ ^61^ ^]^ Copyright 2019, American Chemical Society. h) Fabrication process and SEM image of the PAM/graphene hydrogel. Reproduced with permission.^[^ ^83^ ^]^ Copyright 2021, Elsevier. i) Schematic showing the internal structure of hydrogel. Reproduced with permission.^[^ ^84^ ^]^ Copyright 2021, American Chemical Society. j) Schematic of the preparation of the PDA‐CNTs/PVA hydrogel. Reproduced with permission.^[^ ^86^ ^]^ Copyright 2021, American Chemical Society. k) Schematic diagram of the PSGP hydrogel. Reproduced with permission.^[^ ^87^ ^]^ Copyright 2020, American Chemical Society. l) Schematic diagram of the synthesis and internal connection of AgNWs/CS‐based hydrogels. Reproduced with permission.^[^ ^60^ ^]^ Copyright 2019, Elsevier. m) Schematic of the fabrication process of the BC/ZnO nanocomposite hydrogel treated with amino‐silane. Reproduced with permission.^[^ ^71^ ^]^ Copyright 2020, American Chemical Society. n) Mechanism for enhanced cohesiveness of the catechol‐chitosan‐diatom hydrogel. Reproduced with permission.^[^ ^89^ ^]^ Copyright 2021, Elsevier.

This review focuses from this point onwards on single network hydrogels that are less common. As the main component in the mammalian extracellular matrix, a pure hyaluronic acid (HA) hydrogel was developed by Kim et al.^[^ ^75^ ^]^ **Figure** **5**a presents the fabrication process of the HA hydrogel film, in which 1,4‐butanediol diglycidyl ether serves as the crosslinker of the HA films. Intriguingly, the HA hydrogel film is regarded as the triboelectric material in TENGs, which differs from most other studies that normally consider hydrogels as electrodes. Another interesting work was reported by Chang et al.,^[^ ^76^ ^]^ where an egg white hydrogel (EWH) made from egg white and the egg white liquid (EWL) were utilized as electrodes in TENGs. The macroscopic phase transition of EWH in Figure 5b can be observed by vertically stacking and sealing two EWHs dyed with red and green fluorophores. As time elapses, the two separated hydrogels fuse by gravity and a steady network is generated when the egg white solution is transferred to EWH. Self‐liquidation from EWH to EWL occurs during the phase transition which is synchronized with EHW and alkali generation.

In comparison with the single network hydrogels, double network hydrogels have gained more attention in recent years because of the advantageous mechanical and electric properties. Instead of focusing on these general two‐polymer or co‐polymer structures,^[^ ^77^, ^78^, ^79^ ^]^ our discussion focuses on identifying more complex/unique compositional examples. For instance, Panwar et al.^[^ ^51^ ^]^ have synthesized a carboxymethyl chitosan (CMCh) /carboxymethyl cellulose/PDA (Ch‐CMC‐PDA) hydrogel by combining CMCh and CMC‐D‐PDA based on physical and covalent interactions, as shown in Figure 5c. The pre‐formation of CMC‐D‐PDA proceeds by two steps: introduction of aldehyde groups by CMC oxidation and polymerization of dopamine. The covalent interaction between the aldehyde and amine groups on CMCh generated reversible dynamic imine bonds. Sun et al.^[^ ^80^ ^]^ have proposed a physically and chemically cross‐linked double network hydrogel containing poly(3,4‐ethylenedioxythiophene):poly(styrene sulfonate) (PEDOT:PSS), gelatin, and PAM. As shown in Figure 5d, the gelatin and acrylamide were dissolved in water and after introduction of PEDOT:PSS, polymerization of PAM with linear gelatin took place. Interestingly, after cooling to 4 ℃, the triple helix structure of gelatin was formed by hydrogen bonding.

### 4.2 Salt‐Modified Hydrogels

Salts enhance the ionic conductivity of hydrogels and synthesis of salt‐modified hydrogels is therefore quite straightforward. Addition of salts normally shows no negative effect on the transmittance and mechanical properties of hydrogels. There have been many studies using inorganic salts to produce ionic conductors in TENGs.

As shown in Figure 5e, Qi et al.^[^ ^81^ ^]^ have introduced LiCl salts into single network PAM hydrogels. It is interesting that the dissolved Li^+^ and Cl^−^ interact with water molecules in the hydrogels to form hydrated ions. Unlike free water molecules, a larger amount of energy is required to evaporate water solvates ions. LiCl can therefore be used to reduce the drying rate of hydrogels. By adopting the same mechanism and similar strategies, inorganic salts—such as NaCl, KCl, and CaCl_2_—are often added into different hydrogels during H‐TENG fabrication.

Apart from the formation of hydration ions, the added salts in PAM/PVA hydrogels have been reported to play more essential roles. Wu et al.^[^ ^63^, ^82^ ^]^ have described the salting‐out phenomenon caused by the Hofmeister effect that enabled hydrogels to form intermolecular hydrogen bonds; the electrostatic interaction resulted in the formation of sodium/lithium bonds. Thus, Na^+^ and Cl^−^ ions interacting with polymer chains ( Figure 5f) may produce a stable channel for charge transfer. According to the Hofmeister effect, NaCl causes the salting‐out phenomenon, enabling hydrogels to have better mechanical properties and stability; while sodium bonds that are stronger than hydrogen bonds can be formed by electrostatic interaction. Based on the similar strategy albeit different mechanism, sodium tetraborate has been introduced to the PVA/sodium alginate (SA) hydrogels followed by immersion in the CaCl_2_ solution, as shown in Figure 5g.^[^ ^61^ ^]^ Boronic ions aid the formation of a borax cross‐linked PVA network, whereas Ca^+^ ions generate a calcium cross/lined alginate structure. Combining with these additional dynamic cross‐links, a robust double network structure with enhanced conductivity was constructed to enhance the performance of H‐TENGs.

### 4.3 Carbon‐Modified Hydrogels

Carbon‐based materials—such as graphene and carbon nanotubes (CNTs)—are also common conductive additives for hydrogels. Carbon‐based materials are used for two main reasons: i) most carbon‐based materials are 1D or 2D materials with large aspect ratios, thus improving the mechanical strength of the hydrogels; and ii) similar to salts, carbon‐based materials facilitate charge transfer during the contact‐separation cycles, which enhances the output performance of H‐TENGs. However, in spite of the enhanced mechanical properties and conductivity, carbon‐modified hydrogels often lose the transparency and health safety.

Carbon‐based materials are notoriously difficult to disperse evenly in hydrogels. Chen et al.^[^ ^83^ ^]^ were able to overcome this problem by combining the hydrogel precursor with a graphene aqueous dispersion as shown in Figure 5h. Incorporation of 5 wt% graphene into the PAM hydrogel generated a 3D network, which offered conductive pathways for charge transfer and current collection. Based on the same purpose, Yang et al.^[^ ^84^ ^]^ have introduced a PDA network on CNT surfaces by spontaneous oxidation and polymerization of dopamine in a weakly alkaline medium to enhance the dispersion of PDA‐CNTs in the hydrogels. As shown in Figure 5i, the PDA‐CNTs and PVA were further mixed with Ba_2_B_4_O_7_ to generate dynamic borate bonds. Thus, a PVA‐based hydrogel with an even dispersion of PDA‐CNTs was obtained. Alternatively, MXene—a 2D transition metal carbide nanomaterial—has been used analogously as carbon‐based additives to PVA hydrogels.^[^ ^85^ ^]^ The large specific surface area and unique composition of MXene, when used in the hydrogel composition, promote the stretchability and conductivity of the hydrogels.

It is also common to add carbon‐based materials into the double or multi network hydrogels. He et al.^[^ ^86^ ^]^ have incorporated graphene oxide (GO) and PEDOT:PSS into PAM/silk fibroin (SF) hydrogels. Figure 5j illustrates the formation of a stable hydrogel network with GO sheets intertwined into the PAM and SF networks, leveraging the GO hydrogen bonding capabilities. It is noted that both GO and PEDOT:PSS increase the conductivity and tensile strain of the hydrogel. Similarly, Liu et al.^[^ ^87^ ^]^ have added graphene to a PVA‐based hybrid hydrogel as shown in Figure 5k. Graphene improves not only the porosity of the structure and tensile strength of the hydrogels, but also the conductivity to enhance the device output.

### 4.4 Other Hydrogels

Other hybrid hydrogels containing additives such as metals, clays, and inorganic oxides, have also been reported as electrodes and conductors in H‐TENGs. For example, the inorganic nanoclay has been added to the polyzwitterion hydrogel to increase mobile ions and impart reversible cross‐linking improving the toughness of the hydrogels.^[^ ^88^ ^]^

As shown in Figure 5l, Wang et al.^[^ ^60^ ^]^ have mixed the Ag NW suspension and aqueous chitosan solution to form AgNW‐embedded chitosan. After casting the chitosan/Ag NW solution on a glass slide, the AgNO_3_ or CuSO_4_ solution was placed in the chitosan/Ag NW mixture to produce crosslinking by generating an interwoven structure via complexation of functional groups (such as —OH and —NH_2_) of the chitosan chains and Ag^+^ or Cu^2+^ cations. The composite hydrogels based on hybrid ionic and electronic conduction are effective current collectors in TENGs for biomechanical energy harvesting. Meanwhile, Ag NWs have also been reported in mixtures with PVA hydrogels to avoid the unevenness and brittleness of the hydrogels while improving the conductivity of PVA hydrogels.^[^ ^54^ ^]^

Alternatively, Jakmuangpak et al.^[^ ^71^ ^]^ have introduced ZnO nanoparticles into a bacterial cellulose (BC) hydrogel. As shown in Figure 5m, the BC hydrogel was immersed in the ZnCl_2_ solution and soaked in NaOH at 55 ℃. Under continuous stirring, ZnO was formed on the BC hydrogel by the chemical reaction between ZnCl_2_ and NaOH. To ensure strong adhesion between the BC/ZnO film and ITO substrate, the nanocomposite was immersed in the amino‐silane solution to modify the surface energy of the BC/ZnO film and produce good adherence to the substrate. The BC/ZnO film has obvious clusters of ZnO nanoparticles, which impacts the triboelectric effect and electrostatic induction in TENGs. As shown in Figure 5n, Kim et al.^[^ ^89^ ^]^ have directly added pill‐shape diatom frustules to catechol chitosan. The catechol‐chitosan‐diatom hydrogel showed increased cohesiveness, because the added diatom frustule enhanced the gel integrity and shape maintenance caused by physical–chemical entanglement with various functional groups of the polymer matrix.

## 5 Advanced Functions of H‐TENGs

Unlike other flexible electrodes, hydrogels have some unique functions such as self‐healing for applications under extreme conditions and mechanical reliability for device integrity and charge transfer. To solve the easy drying drawback of common hydrogel electrodes in H‐TENGs, advanced functions such as temperature tolerance and gel properties are also introduced.

### 5.1 Self‐Healing

Although the excellent flexibility and stretchability of hydrogels are desirable for flexible and wearable electronics, their relatively poor mechanical strength is a weakness compromising the long‐term stability and working environments. Therefore, researchers have recently focused on developing hydrogels—and their resulting H‐TENGs—with the self‐healing functionality.^[^ ^49^, ^51^, ^61^, ^89^ ^]^ To achieve self‐healing, most studies have introduced additional dynamic functional groups and/or bonds into hydrogels and triboelectric materials.

**Figure 6:**
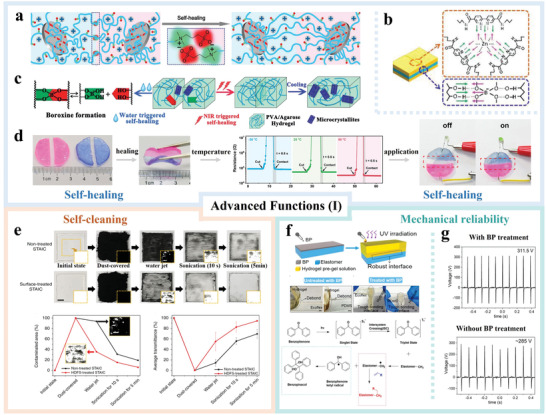
Advanced functions (I) of hydrogels in TENGs: a) schematic illustration of cut hydrogels healed by electrostatic attraction between polyzwitterions and rebuilding polyzwitterion–clay adsorption. Reproduced with permission.^[^ ^88^ ^]^ Copyright 2020, Wiley‐VCH. b) Schematic of the self‐healing mechanisms of EHTS‐TENG. Reproduced with permission.^[^ ^90^ ^]^ Copyright 2019, Wiley‐VCH. c) Self‐healing mechanisms of the PVA/PDAP/MWCNT hydrogel upon exposure to NIR and water. Reproduced with permission.^[^ ^92^ ^]^ Copyright 2019, Royal Society of Chemistry. d) Photographs of the self‐healing process and current connection for glycerol‐based PAAM‐Clay organohydrogel. Reproduced with permission.^[^ ^62^ ^]^ Copyright 2021, Elsevier. e) Self‐cleanability of HDFS‐treated STAICs explored by cleaning surfaces contaminated by activated charcoal powders. Reproduced with permission.^[^ ^59^ ^]^ Copyright 2018, Springer Nature. f) Schematics of a practical fabrication approach for robust hydrogel elastomer hybrid. Photographs and mechanism of elastomer/hydrogel interfaces with or without BP treatment. Reproduced with permission.^[^ ^77^ ^]^ Copyright 2018, American Chemical Society. g) *V*_OC_ of DH‐TENG with or without BP modification. Reproduced with permission.^[^ ^96^ ^]^ Copyright 2020, Royal Society of Chemistry.

Gao et al.^[^ ^88^ ^]^ have prepared a self‐healing polyzwitterion‐clay nanocomposite hydrogel through noncovalent bonding (detailed mechanism is shown in **Figure** **6**a). When two freshly cut hydrogel sections are put in contact, the accessible polymer chains at the hydrogels surface form electrostatic attraction among the zwitterion groups. This interaction results in quick interfacial adhesion and rebuilding of ionic channels. Consequently, bridging of adjacent nanoclays occurs across the interface with further diffusion and tangling of polymer chains to improve the self‐healing efficiency *ƞ* (*ƞ =* *λ*_a_/*λ*_b_, where *λ_b_* and *λ_a_* are the quantified mechanical property values, such as tensile stress and tensile strain, before and after hydrogels undergo self‐healing process, respectively^[^ ^87^ ^]^). Another similar example can be found in Lai et al.’s work,^[^ ^90^ ^]^ where a self‐healing PVA/borax H‐TENG was developed. As shown in Figure 6b, the high self‐healing efficiency of 100% was mainly attributed to the kinetically labile coordination between bipyridine and zinc ions in the PDMS, and the hydrogen bonds between hydroxy groups endowed PDMS/hydrogel and B(OH)^−^_4_ in the hydrogel. As a result, the H‐TENG showed almost the same mechanical and electrical properties after healing for 30 min.

Apart from healing efficiency, the healing time and temperature are another two factors that affect the self‐healing properties of hydrogels. For example, it takes two hours for the polyacrylic acid/nanochitin composite hydrogel to achieve a self‐healing efficiency of 97% based on the formation of dynamic metal‐coordination bonds between aluminum ions and carboxyl groups.^[^ ^64^ ^]^ This dynamic process is time consuming considering that TENGs undergo continuous contact and separation during mechanical energy harvesting. In this respect, Shuai et al.^[^ ^91^ ^]^ have developed a self‐healing hydrogel with a high healing efficiency of 85% after only 10 min healing, but the healing temperature was as high as 60 ℃. A high temperature is not preferred in practice, which makes the hydrogels easier to lose water.

Recently, Yang et al.^[^ ^84^ ^]^ have prepared a self‐healing device that could recover from mechanical damage after healing for 10 min at room temperature by taking advantage of the borate ester bonds in the hydrogels and dynamic imine bonds in the triboelectric layer. The chemical self‐healing process takes place with the help of water through the formation of dynamic and reversible covalent crosslinking borate bonds. Guan et al.^[^ ^92^ ^]^ have produced a self‐healing H‐TENG, both mechanical and electrical properties of which could recover in 1 min under near‐infrared (NIR) light. The detailed healing mechanism presented in Figure 6c, indicating that both water and NIR trigger the self‐healing in the hydrogel. A physical self‐healing process was concluded to occur under NIR irradiation, and continuous reorganization of the molecular chains was caused by recrystallization of the PVA hydrogels in the reversible process. A similar report with a healing efficiency of 98% after exposure for 1 min to air has been reported by Liu et al.^[^ ^87^ ^]^ Intriguingly, Huang et al.^[^ ^62^ ^]^ have developed a self‐healable PAM/clay H‐TENG with excellent recovery ability in a wide temperature window. As shown in Figure 6d, the obtained organohydrogel (i.e., a hydrogel containing organic solvent) healed in 1 second from −30 to 80 ℃, maintaining the same resistance, mechanical properties, and electrical output. Therefore, when connecting two damaged sections together in a circuit at a voltage of 5 V, a LED bulb was powered.

### 5.2 Self‐Cleaning

Unlike self‐healing, self‐cleaning is a function that is common for regular TENGs but not for H‐TENGs. However, hydrogels and triboelectric layers can be contaminated easily. The contaminated TENGs usually show lower output due to the decreased surface charge density and therefore, it is important to incorporate H‐TENGs with the self‐cleaning capability.

Up to date, there is only one study on the self‐cleaning property of H‐TENGs. Lee et al.^[^ ^59^ ^]^ have developed a self‐cleanable, transparent, and attachable ionic communicator (STAIC) by chemical functionalization of the PDMS surface. As shown in Figure 6e, no obvious difference was observed for STAIC with or without the surface treatment in the initial and dust‐covered states. However, after water jetting or sonicating for different duration times, the contaminated area on the untreated STAIC was almost twice of that on the surface‐treated STAIC. Conversely, the average transmittance of the untreated STAIC is much lower than the functionalized STAIC, indicating the effectiveness of the surface treatment in the self‐cleaning function.

### 5.3 Mechanical Reliability

In the fabrication of common TENGs, metal electrodes are usually attached to the triboelectric materials,^[^ ^93^, ^94^, ^95^ ^]^ for better charge induction and transfer during the contact and separation cycles. However, most H‐TENGs have polymer films, particularly PDMS and silicone rubber, as the coverage and triboelectric materials. The mechanical reliability between the relatively hydrophobic polymers and hydrophilic hydrogels worsens, andthus charge induction and device performance are negatively affected. Therefore, improving the mechanical reliability between hydrogels and triboelectric materials is another important task for H‐TENGs.

In 2018, Liu et al.^[^ ^77^ ^]^ have used a BP treatment with the assistance of ultra‐violet (UV) irradiation to build tough interfacial bonding among elastomers and hydrogels as shown in Figure 6f. Compared to the interface without the BP treatment, a robust hydrogel–elastomer interface was observed from the elastomer–hydrogel after the BP treatment. The mechanism of the BP treatment can be explained as follows. Upon exposure to UV light, BP is excited to the singlet state and then triplet state by intersystem crossing. Consequently, the triplet BP state abstracts a hydrogen atom from a polymer in close proximity and generates BP ketyl radicals. The methyl radical then acts as a crosslinker by creating, and subsequently combining, radicals from the two polymers at the interface, in addition to forming benzopinacol as a by‐product.

Since then, many reported H‐TENGs have utilized the same BP chemistry to enhance the mechanical reliability of polymer interfaces.^[^ ^54^, ^59^, ^81^, ^96^ ^]^ Among them, there has been one work directly comparing the output and long‐term stability of H‐TENGs with or without the BP treatment.^[^ ^96^ ^]^ As shown in Figure 6g, the open‐circuit voltage (*V*_OC_) of the PAM/NaCl‐based TENGs after the BP treatment was slightly higher than that without the BP treatment. However, after continuous operation at 10 Hz for 30 min, the output of the BP interfacial treatment remained on the same level, while that of the non‐treated interface decreased significantly. Therefore, the mechanical reliability and longevity improve the potential output stability of these energy harvesting devices along with output enhancement.

### 5.4 Temperature Tolerance

Despite getting increasing attention as ionic conductors in recent years, hydrogels are susceptible to drying and freezing, thus hampering applications at high and low temperatures and the resulting lifetime. Hence, it is necessary to improve the temperature tolerance of hydrogels, and H‐TENGs are no exception.

**Figure 7:**
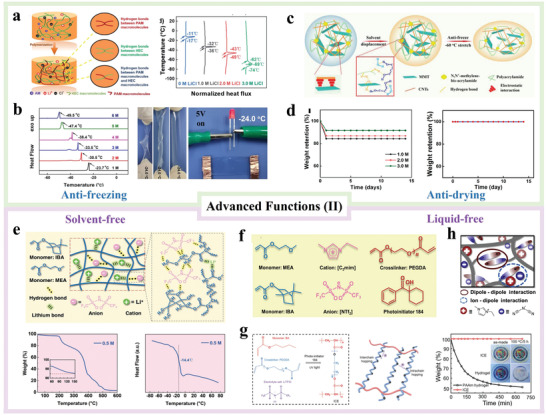
Advanced functions (II) of hydrogels in TENGs: a) schematic illustration of the polymerization process and DSC curves of PAM/HEC/LiCl hydrogels. Reproduced with permission.^[^ ^97^ ^]^ Copyright 2020, Royal Society of Chemistry. b) DSC curves and photograph of flexibility and conductive properties of cellulose/NaCl hydrogel films. Reproduced with permission.^[^ ^98^ ^]^ Copyright 2021, Elsevier. c) Schematic fabrication of the environmentally tolerant PAM/MMT/CNTs organohydrogel. Reproduced with permission.^[^ ^100^ ^]^ Copyright 2021, Wiley‐VCH. d) Weight change of cellulose/NaCl organohydrogel and TENGs. Reproduced with permission.^[^ ^101^ ^]^ Copyright 2021, American Chemical Society. e) Schematic illustration of the molecular design of the liquid‐free P(MEA‐co‐IBA)/LiTFSI elastomer and TGA and DSC curves. Reproduced with permission.^[^ ^103^ ^]^ Copyright 2021, Wiley‐VCH. f) Molecular structures of elements for preparing ionogels. Reproduced with permission.^[^ ^79^ ^]^ Copyright 2021, Wiley‐VCH. g) Molecular structure of the PBA/PEGDA/LiTFSI gel, mechanism of ion transport, weight change of the hydrogel, and TENGs. Reproduced with permission.^[^ ^104^ ^]^ Copyright 2020, Wiley‐VCH. h) Schematic of the ion and dipole interactions in the PAA/PDMAPS ionogel. Reproduced with permission.^[^ ^105^ ^]^ Copyright 2019, Elsevier.

There are several methods to address the temperature working range of H‐TENGs; the common technique is to introduce inorganic salts within the hydrogels to decrease the freezing point of the water swollen inside the gel. For example, Bao et al.^[^ ^97^ ^]^ have added LiCl with different concentrations to PAM/hydroxyethyl cellulose hydrogels as shown in **Figure** **7**a. For pure hydrogels, the phase transition point of water by DSC was around −11 ℃. This was a much higher freezing point compared to a hydrogel with LiCl addition, where the lowest freezing point was down to −62 ℃ (3 M LiCl). Such a phenomenon arises from the colligative properties of dissolved LiCl leading to decrease in the water vapor pressure and additionally, a reduction of the solution freezing point. Similar strategies have been adopted by Hu et al.^[^ ^98^ ^]^ who added different amounts of NaCl to cellulose hydrogels. When the salt concentration was increased from 1 to 6 m, an obvious decrease in the freezing point from −23.7 to o −49.5 ℃ was observed in Figure 7b. As a result, the developed anti‐freezing hydrogel was able to maintain the flexibility, stretchability, and conductivity at −24 ℃.

While salts are capable of decreasing the freezing point, they do not inhibit water evaporation from hydrogels at high temperature. Until 2018, a simple one‐pot solvent replacement method has been proposed by Chen et al.^[^ ^99^ ^]^ By immersing Ca‐alginate/PAM hydrogels in cryo solutions, the non‐drying and anti‐freezing organohydrogel not only maintained the flexibility and transparency at −70 ℃, but also retained the original weight after storage for 15 days at 20 ℃.

Inspired by this strategy, Sun et al.^[^ ^100^ ^]^ have fabricated PAM/montmorillonite/CNTs organohydrogels as shown in Figure 7c. The samples exhibited excellent temperature tolerance in the range of −60–60 ℃ and good stability retaining the conductivity and stretchability after storage for 30 days under ambient conditions. Consequently, the corresponding H‐TENGs showed a stable output at −60–60 ℃. On the other hand, Qian et al.^[^ ^101^ ^]^ soaked cellulose hydrogels in a glycerol/NaCl/water solution to obtain a high conductivity of 0.72 S m^−1^ that was beneficial to the sensing and energy harvesting of TENGs. The organohydrogels and H‐TENGs both showed very stable weights after storage for 15 days at room temperature (Figure 7d). Similarly, Wang et al.^[^ ^102^ ^]^ have immersed PVA/hydroxyethyl cellulose (HEC) hydrogels in salt/glycerol/water solution. The obtained organohydrogels showed both high conductivity of 5.77 S m^−1^ and excellent temperature tolerance of −35–65 °C.

### 5.5 Gel Properties

The major problem of the cryoprotecting displacement method is the non‐negligible conductivity decrease of the organohydrogel after the immersion process. Alternative approaches to adding inorganic salts to aqueous‐based gels exist and focus on exchanging water with a higher boiling point polar protic solvent. Furthermore, there have been a few recent studies on the fabrication of gels without water or volatile solvents. Apart from the above‐mentioned advantages (such as transparency, stretchability, tunable conductivity, etc.) of common hydrogels, these water‐free gels also show merits of longer performance stability (months, compared to days of hydrogels) and higher conductivity (as no solvent replacement is needed, compared to organohydrogels). Given the same synthesis mechanism and similar properties, the development of gel‐based TENGs can be considered as one of the advanced technologies/functions to expand the practical applications of H‐TENGs.

Yiming et al.^[^ ^103^ ^]^ have dissolved lithium bis(trifluoromethanesulfonyl)imide (LiTFSI) in the liquid monomer mixture of ethylene glycol methyl ether acrylate (MEA) and isobornyl acrylate (IBA). Figure 7e illustrates the formation of a liquid‐free crosslinked P(MEA‐co‐IBA) ionogel, in which lithium bonds and hydrogen bonds are formed between LiTFSI and polymer chains. Upon application of an electric field, lithium ions are able to move among the polymer chains resulting in high ionic conductivity of the ionogel. The developed alternative solvent H‐TENGs showed potentially excellent outputs and a wide working temperature range from −14.4 to 200 °C. The same group have also used ionic liquids to prepare stable ionogels that have the potential to reach higher temperatures not available with volatile solvents due to the vapor pressure.^[^ ^79^ ^]^ The detailed molecular structures of these materials are shown in Figure 7f. Results with high conductivity and a wide operating temperature window (−60–200 °C) have been observed in the area of solvent‐free ionogels.

Another liquid monomer (BA) has been used as the solvent to dissolve the LiTFSI salt.^[^ ^104^ ^]^ Figure 7g shows the ion transport mechanism of the ionic elastomer, in which the intrachain or interchain hooping inside the polymer chains contributes to the ion transport. In comparison with the PAM hydrogel that underwent a 50% weight loss after storing at 100 °C for 5 h, the ionic elastomer maintained the original weight under the same conditions. Moreover, the corresponding TENG showed a stable output after storage for 5 months at room temperature. On the other hand, Sun et al.^[^ ^105^ ^]^ have prepared ionogels by dissolving acrylic acid and 3‐dimethyl (methacryloyloxyethyl) ammonium propane sulfonate monomers in the ionic liquid ([EMI][DCA]). The dipole‐dipole and ion‐dipole interactions shown in Figure 7h contributed to the formation of the ionogel network. Compared to the same H‐TENG that used water as the solvent—which stoped working after 48 h—the ionogel showed a wide working temperature window (−20–100 °C) and the TENG maintained a stable *V*_OC_ even after storage for 1 month in a dry environment.

## 6 Enhanced Outputs of H‐TENGs

To further improve the output of H‐TENGs, several strategies including conductivity enhancement, surface modification, two‐electrode mode, and counterpart optimization have been introduced as summarized in **Table** **1** and discussed in detail as follows.

**Table 1:** Output performance comparison of reported H‐TENGs based on various improvement methods

Method	Hydrogel	Tribo‐pair	Size [cm^2^]	*V*_OC_ [V]	*I*_SC_ [mA m^−2^]	Ref
Conductivity enhancement	PVA/PDAP/CNT	SR^(1)^‐skin	3 × 3	70→95	0.89→1.24	^[^ ^92^ ^]^
	CS^(2)^/Ag NWs/Cu	PDMS‐skin	2 × 2	174→218	25→34.4	^[^ ^60^ ^]^
	PSGP^(3)^	PSGP‐PDMS	2 × 2	12	0.50	^[^ ^86^ ^]^
	PAM/HEC/LiCl	SR‐skin	3 × 3	285	17.2	^[^ ^97^ ^]^
	P(MEA‐IBA)/LiTFSI	VHB‐latex	4 × 1	3	0.75	^[^ ^103^ ^]^
	Cellulose/NaCl	VHB‐glove	3 × 3	187	0.57	^[^ ^98^ ^]^
	PVA/PDAP/graphene	PDMS/CNT‐Cu	2.5 × 2.5	69→132	/	^[^ ^87^ ^]^
	PAM/PVA/NaCl	Ecoflex‐skin	2 × 2	90→245	2.6→6.2	^[^ ^63^ ^]^
Two‐electrode mode	PAM/LiCl	PDMS‐Nylon (Al)	3 × 4	145→182	1.25→16.67	^[^ ^28^ ^]^
	PAM/alginate	PDMS‐skin (Ecoflex)	4 × 1.8	70→100	0.64→0.51	^[^ ^77^ ^]^
	PVA/Na‐alginate	PDMS‐Al (Al)	2 × 5	98.6→203	7.3→17.6	^[^ ^61^ ^]^
	PAM	PDMS‐PBS/PBMA	2.5 × 5	280	2.72	^[^ ^74^ ^]^
	PAM/NaCl	PDMS‐TPU (Al)	5 × 5	113→311	→12.96	^[^ ^96^ ^]^
Surface modification	PVA	PDMS‐Al	8 × 8	200	3.52	^[^ ^27^ ^]^
	PAM/LiCl	PDMS‐Al	4 × 4	252→313	12.5→14.7	^[^ ^59^ ^]^
	PAA/NaCl	Ecoflex/ZnS‐skin	3 × 5	180	43.30	^[^ ^130^ ^]^
	PVA/Ag NWs	Ecoflex‐water/Al	5 × 5	37→78	/	^[^ ^54^ ^]^
	BC^(4)^/ZnO	Teflon‐BC/ZnO	4 × 5	26→31	1.15→1.4	^[^ ^71^ ^]^
	PAM/clay	PDMS/IU‐skin	4 × 4	157	10	^[^ ^62^ ^]^
	Agarose/KCl	PTFE‐CS/glycerol	≈10	80	2.7	^[^ ^117^ ^]^
Counterpart optimization	PVA/PEI	PVA/PEI‐PET (paper)	2.5 × 2.5	2.74→79.6	0.096→8.18	^[^ ^78^ ^]^
	PVA/borax	PDMS‐latex (FEP)	4 × 4	2→21	/	^[^ ^90^ ^]^
	PAM/PDA	Nitrile‐PTFE (VHB)	2.5 × 2.5	31→230	4.8→20	^[^ ^49^ ^]^
	PNA/LiCl	PMA‐PTFE (rubber)	/	10→−36	0.7 µA	^[^ ^91^ ^]^
	Cellulose/NaCl	VHB‐latex (nitrile)	2 × 2	70→120	2.37	^[^ ^101^ ^]^
	PVA/MXene	Ecoflex‐Kapton (Cu)	2 × 5	12→230	0.28	^[^ ^85^ ^]^

Note: (1) SR: silicone rubber; (2) CS: chitosan; (3) PSGP: PAM/silk fibroin/graphene oxide/PEDOT:PSS; and (4) BC: bacterial cellulose.

### 6.1 Conductivity Enhancement

Different from metal electrodes, hydrogel electrodes usually have much lower conductivity. Therefore, it is common to focus on the improvement of conductivity when considering hydrogels in electrical applications.^[^ ^106^, ^107^, ^108^, ^109^, ^110^ ^]^ In particular, the conductivity of hydrogels in TENGs affects charge induction and electron transfer during the contact and separation cycles. Thus, attention has also been paid to improve the conductivity of hydrogels in TENGs.

**Figure 8:**
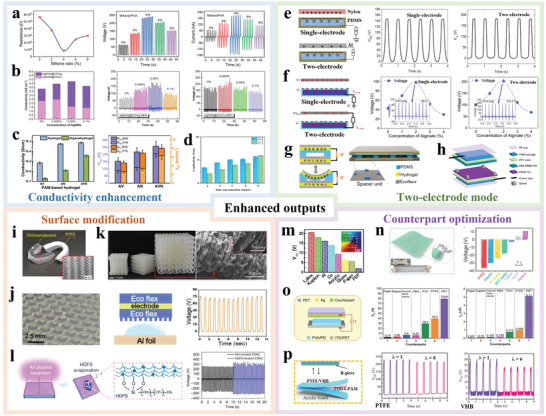
Enhanced outputs of H‐TENGs: a) resistance of MXene/PVA hydrogels and output performance of MH‐TENG. Reproduced with permission.^[^ ^85^ ^]^ Copyright 2021, Wiley‐VCH. b) Conductivity of AgNWs@CS/Ag or Cu hydrogels and *V*_OC_ of the TENGs. Reproduced with permission.^[^ ^60^ ^]^ Copyright 2019, Elsevier. c) Conductivity of PAM‐based hydrogels and *V*_OC_ of the TENGs. Reproduced with permission.^[^ ^63^ ^]^ Copyright 2021, American Chemical Society. d) Conductivity of the PVA‐HEC hydrogels after immersion in salt solutions. Reproduced with permission.^[^ ^102^ ^]^ Copyright 2021, Elsevier. e) Schematic illustration of single‐/two‐electrode modes and *V*_OC_ of PAM/LiCl H‐TENGs. Reproduced with permission.^[^ ^28^ ^]^ Copyright 2017, American Association for the Advancement of Science. f) Schematic illustration of single‐/two‐electrode modes and *V*_OC_ of PAM/SA H‐TENGs. Reproduced with permission.^[^ ^61^ ^]^ Copyright 2019, American Chemical Society. g) Schematic diagram of fabrication of PAM/alginate two‐electrode TENG. Reproduced with permission.^[^ ^77^ ^]^ Copyright 2018, American Chemical Society. h) Schematic of the configuration of HD‐TENG. Reproduced with permission.^[^ ^74^ ^]^ Copyright 2020, American Chemical Society. i) Photograph of the mouthguard‐TENG and SEM image of chitosan/glycerol surface. Reproduced with permission.^[^ ^117^ ^]^ Copyright 2021, Elsevier. j) Photograph of the honeycomb‐shaped PA hydrogel and *V*_OC_ of its TENG on water‐bearing interface. Reproduced with permission.^[^ ^54^ ^]^ Copyright 2019, Elsevier. k) Digital and SEM images of the 3D printed ultraflexible part. Reproduced with permission.^[^ ^118^ ^]^ Copyright 2018, Elsevier. l) Schematic of the surface modification process and *V*_OC_ comparison of its PAM/LiCl H‐TENGs with/without HDFS treatment. Reproduced with permission.^[^ ^59^ ^]^ Copyright 2018, Springer Nature. m) Dependence of *V*_OC_ on the contact materials and photograph of EHTS‐TENG. Reproduced with permission.^[^ ^90^ ^]^ Copyright 2019, Wiley‐VCH. n) Schematic of the PNA/PMA core‐sheath fiber H‐TENG and *V*_OC_ based on various counterparts. Reproduced with permission.^[^ ^91^ ^]^ Copyright 2020, Elsevier. o) Configuration and output performance of the TE‐TENG in the two‐electrode mode with different counterparts. Reproduced with permission.^[^ ^78^ ^]^ Copyright 2019, Wiley‐VCH. p) Schematic and *V*_OC_ of PDA/PAM H‐TENGs with PTFE or VHB films under different stretching conditions. Reproduced with permission.^[^ ^49^ ^]^ Copyright 2020, Royal Society of Chemistry.

The common method for conductivity improvement is to introduce conductive additives to the hydrogels. A typical example can be found in Luo et al.’s work,^[^ ^85^ ^]^ where MXene nanosheets were added to the PVA hydrogel to form microchannels on the surface to improve ion transport. Hence, the resistance of MXene/PVA shown in **Figure** **8**a was smaller than that of pure PVA, even though an excessive amount of additives increases the resistance slightly. Consequently, the *V*_OC_ and *I*_SC_ values of the corresponding TENGs increased initially and then decreased, and the optimal output was achieved after the addition of 4% MXene. Similarly, He et al.^[^ ^86^ ^]^ have added GO and PEDOT:PSS to the PAM/SF hydrogel to improve the conductivity and an interwoven structure composed of chitosan and metal ions was formed.^[^ ^60^ ^]^ As shown in Figure 8b, adding different concentrations of Ag NWs into metal ion‐bonded hydrogels produced different conductivities. The overall conductivity of the Cu‐bonded hydrogel was lower than that of the Ag‐bonded hydrogel, while the output of the former was higher than that of the latter. It is mainly because the conductivity was dominated by ion transfer in the former but electron transfer in the latter.

Given the emergence of organohydrogel‐based TENGs with excellent anti‐freezing and non‐drying properties, the conductivity enhancement of organohydrogels has also been studied recently. Wu et al.^[^ ^63^ ^]^ have proposed the double network structure with sodium bonds based on the electrostatic interaction to construct stable charge channels in the organohydrogel. As a result, the conductivity of the PAM/PVA/NaCl organohydrogel in Figure 8c was much higher than that of the PAM/NaCl and PAM/PVA organohydrogel. Consequently, the *V*_OC_ and *J*_SC_ values of the corresponding TENG were also larger than those without double network and/or sodium bonds. On the other hand, Wang et al.^[^ ^102^ ^]^ have immersed PVA/HEC hydrogels in the salt glycerol/water solution. As shown in Figure 8d, different kinds and concentrations of salt solutions resulted in different conductivities. Considering that the conductivity increased with the salt concentration, the immersion method is also effective in enhancing the conductivity of hydrogels.

### 6.2 Two‐Electrode Mode

When utilizing hydrogel as the electrode in TENGs, the most common configuration is the single‐electrode mode because of the simple fabrication and wearable feasibility. However, according to the structural figure‐of‐merit (especially for TENGs), the output of two‐electrode TENGs is much higher than that of single‐electrode TENGs under the same fabrication and measurement conditions. This phenomenon occurs because of the limited transfer charges and suppressed built‐in voltage of the single‐electrode working mode.^[^ ^111^, ^112^ ^]^ Therefore, some researches have introduced the two‐electrode working mode into H‐TENGs for their potential output enhancement.

For example, Pu et al.^[^ ^28^ ^]^ have fabricated a single‐electrode PAM/LiCl H‐TENG with elastomer films as the covering and triboelectric materials. As shown in Figure 8e, the optimal performance of the single‐electrode TENG with nylon and PDMS as the triboelectric pair was ≈145 V for *V*_OC_ of 47 nC for Q_SC_. These values were lower than 182 V and 130 nC of the two‐electrode mode, even though the Al foil has a smaller ability to lose electrons than the nylon film. To better compare the impact of single‐ and two‐electrode modes on the output, Jing et al.^[^ ^61^ ^]^ have chosen the same triboelectric pair (PDMS and Al) for both modes as shown in Figure 8f. It was observed that the device *V*_OC_ of the two‐electrode mode was twice as large as that of the single‐electrode mode, in spite of the same tendency with different alginate concentrations.

Such an obvious output improvement inspired this group to consider PAM/NaCl hydrogel as the electrode for both sides of the two‐electrode mode TENGs. In this case, simple contact and separation of TPU and PDMS films could generate a high *V*_OC_ of 311.5 V, compared to 113.4 V for the Al‐PDMS pair and −36.8 V for the Al‐TPU pair in the single‐electrode mode. Similar strategies using hydrogels as both electrodes in two‐electrode TENGs have been reported in other studies. For instance, Liu et al.^[^ ^77^ ^]^ have used PDMS as one of the triboelectric materials and spacer unit (Figure 8g). During contact and separation between the PDMS and Ecoflex films with a small electron affinity difference, the output voltage of 100 V was still higher than that of 70 V for the single‐electrode even for a skin‐PDMS pair with a large electron affinity difference. Meanwhile, Mi et al.^[^ ^74^ ^]^ have fabricated a two‐electrode TENG with the PAM hydrogel as both electrodes as shown in Figure 8h. A high output of *I*_SC_ at 34 µA and *V*_OC_ at 280 V are obtained corroborating the positive impact of two‐electrode design on the output of H‐TENGs.

### 6.3 Surface Modification

As one of the most popular technologies, surface modification is widely used to improve the output of different types of TENGs. Typically, it is divided into two categories, namely physical modification and chemical functionalization. The former is often used to increase the effective contact between the triboelectric pair, and the latter enhances the charge capturing capability on the surface.

Physical modification techniques such as dry etching,^[^ ^113^ ^]^ 3D printing,^[^ ^114^ ^]^ and particle introduction^[^ ^115^, ^116^ ^]^ have the potential to impact the performance of H‐TENGs. For example, Xu et al.^[^ ^27^ ^]^ have used an Al foil with an anodized nanostructure as the triboelectric counterpart to increase the contact area with H‐TENG. In contrast, Jakmuangpak et al.^[^ ^96^ ^]^ have introduced ZnO nanoparticles into the bacterial cellulose hydrogel to synthesize a rough triboelectric film. An apparently enhanced output was obtained compared to that without ZnO addition.

As shown in Figure 8i, a mouthguard TENG consists of chitosan/glycerol and PTFE films as the triboelectric materials.^[^ ^117^ ^]^ To enhance the output, the chitosan/glycerol precursor was coated on a droplet‐shaped silicon mold and dried overnight. A similar operation has been adopted by Zhang et al.’s study,^[^ ^104^ ^]^ where silicone rubber was casted on a triangular mold to improve the sensitivity to human motions. Another interesting work worthy to mention is that, Qian et al.^[^ ^54^ ^]^ have observed that the honeycomb surface (Figure 8j) did not improve the characteristics of H‐TENGs, but increased the output by two times on a water‐bearing interface. Unlike these above reports, Chen et al.^[^ ^118^ ^]^ have performed 3D printing to fabricate H‐TENGs and in the printed ultraflexible component with rough microstructures (Figure 8k), hydrogels with 3D printed shell were well matched inside this part and thus effective contact and separation could be accomplished.

Different from physical modification, chemical functionalization is capable of introducing some functional groups (such as amine and fluoro) on the triboelectric surface to improve surface charge density.^[^ ^119^, ^120^, ^121^ ^]^ This method has also been applied to H‐TENGs. As shown in Figure 8l, an air plasma was employed to incorporate a small amount of (heptadecafluoro‐1,1,2,2‐tetrahydrodecyl)trichlorosilane into the PDMS surface.^[^ ^59^ ^]^ An obvious increase of 24.5% in *V*_OC_ and 17.5% in *I*_SC_ was obtained from the H‐TENG with HDFS treatment, compared to those without HDFS treatment. The improved result was stemmed from the fluorine layer on PDMS has larger electron binding energy than pure PDMS. However, the fluorine functionalization often requires an air plasma pre‐treatment to generate —OH groups on the substrate surface, which potentially limits the scalability.

### 6.4 Counterpart Optimization

Although empirical triboelectric series have been developed and verified by both theoretical and experimental methods,^[^ ^122^, ^123^, ^124^, ^125^, ^126^ ^]^ there are differences between reports for different materials. Since a perfectly matched triboelectric pair can significantly enhance the device output, the counterpart in H‐TENGs has been studied and optimized in many reported works.

As shown in Figure 8m, a flexible and transparent H‐TENG was fabricated and the device outputs according to the contact materials were summarized, following a decreasing order of Latex > Kapton > Al > Cu > Acrylic > Glass > Paper > FEP.^[^ ^90^ ^]^ This order is different from that of the classical triboelectric series, as Kapton and paper usually are considered as more negative and positive materials, respectively. Other studies confirm the order of this series, such as Shuai et al.,^[^ ^91^ ^]^ fabrication of a H‐TENG woven with poly(NAGA‐co‐AM)/poly(methyl acrylate) (PNA/PMA) core‐sheath fibers (Figure 8n). The *V*_OC_ results indicated that Kapton had a large capability to gain electrons and it was only lower than that of PTFE. Optimization of the counterparts in PAM/LiCl H‐TENGs revealed that paper is a tribo‐positive material between silk and Al, whereas Kapton is a tribo‐negative material close to PDMS in the triboelectric series.^[^ ^28^ ^]^ The difference stems from the materials and measurement conditions, but all in all, the main trend still follows the classic triboelectric series.

In 2019, Wang et al.^[^ ^78^ ^]^ have prepared a dry PVA/PEI hydrogel film as triboelectric materials. To obtain the optimal counterpart, a two‐electrode mode TENG was fabricated as shown in Figure 8o. Both *V*_OC_ and *I*_SC_ exhibited the following tendency: paper < Kapton < PEN < PVC < PTFE < PET, indicating PET and PVA/PEI had the biggest electron affinity difference. In fact, the triboelectric materials can be chosen in an opposite way. For example, Long et al.^[^ ^49^ ^]^ have investigated the impact of negative triboelectric materials on the PDA‐PAM hydrogel surface in both the original and stretched states. The butyronitrile glove was the positive triboelectric material and as shown in Figure 8p, *V*_OC_ of H‐TENGs covered by PTFE before and after stretching was larger than that of H‐TENGs covered by VHB, indicating PTFE is a better choice when the butyronitrile glove is used as the tapping object.

It is noted that the matching dielectric materials of H‐TENGs, usually considered as the packaging and triboelectric materials, are also important to the output enhancement and flexible/wearable applications. As shown in Table 1, elastic films, such as PDMS, SR, VHB, and Ecoflex, have often been considered as the matching dielectrics for H‐TENGs for the following reasons: i) these elastics show large electron affinity difference with textile materials and human skin, which is good for wearable applications; ii) these elastics are usually stretchable, benefiting the flexible activities of human beings, such as bending and twisting; and iii) these elastics are flexible and non‐toxic, thus ensuring the safety and comfortability for portable and wearable electronics. Therefore, elastic dielectrics with non‐toxicity, high flexibility, and strong electron‐gaining ability are ideal for high‐output H‐TENGs in flexible and wearable applications. In fact, elastic materials with the aforementioned properties are also desirable and used in common wearable TENGs.^[^ ^127^, ^128^, ^129^ ^]^

## 7 Flexible and Wearable Applications of H‐TENGs

Similar with other flexible TENGs, H‐TENGs are commonly used in self‐powered sensing/monitoring and electronics powering/charging. More importantly, they can also be applied to other flexible and wearable applications such as human–machine interactions and biomedical electronics.

### 7.1 Self‐Powered Sensing/Monitoring

Nowadays, sensors are becoming more prevalent in our daily lives; this technology is being applied in technical areas such as medicine, robotics, and artificial intelligence. Similar to common flexible TENGs, H‐TENGs are widely used in different types of sensors for wearable applications such as strain sensing,^[^ ^101^ ^]^ temperature sensing,^[^ ^60^ ^]^ and human motions monitoring.^[^ ^84^ ^]^

**Figure 9:**
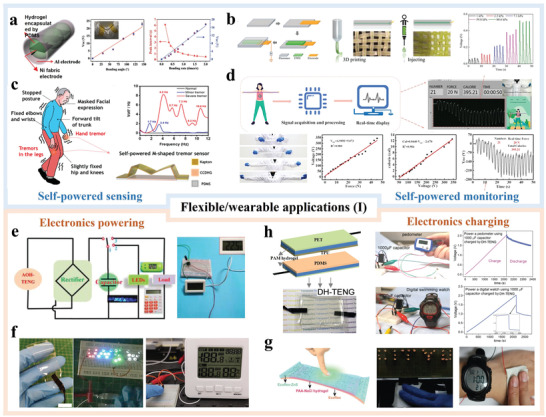
Flexible and wearable applications (I) of H‐TENGs: a) schematic structure of the tube‐shaped TENG and *V*–*t* curves as elbow joint motor sensors. Reproduced with permission.^[^ ^27^ ^]^ Copyright 2017, Wiley‐VCH. b) Schematic of fabrication of the bulk TENG and *V*–*t* curve as a pressure sensor. Reproduced with permission.^[^ ^76^ ^]^ Copyright 2018, Wiley‐VCH. c) Typical symptoms of Parkinson's disease and power spectral density of voltage signal from tremor sensor with various motions. Reproduced with permission.^[^ ^89^ ^]^ Copyright 2021, Elsevier. d) Real‐time demonstration in the actual application of the SA‐Zn‐TENG smart training band and applications. Reproduced with permission.^[^ ^131^ ^]^ Copyright 2021, American Chemical Society. e) Equivalent circuit diagram of the AOH‐TENG based self‐charging power system and photograph showing driving of various electronics. Reproduced with permission.^[^ ^100^ ^]^ Copyright 2020, Wiley‐VCH. f) Photograph of the fabricated SPE‐TENG and applications to LEDs and temperature/humidity meter. Reproduced with permission.^[^ ^132^ ^]^ Copyright 2021, Elsevier. g) Structural configuration of the smart skin device and applications to LEDs and an electronic watch. Reproduced with permission.^[^ ^130^ ^]^ Copyright 2019, Wiley‐VCH. h) Schematic diagram and photograph of the DH‐TENG and applications to a pedometer and digital watch, Reproduced with permission.^[^ ^96^ ^]^ Copyright 2020, Royal Society of Chemistry.

As shown in **Figure** **9**a, a tubular H‐TENG is first developed with electrodes composed of Ni fabric and Al that are deposited inside and outside PVA/PDMS.^[^ ^27^ ^]^ When the H‐TENG was put on the elbow to monitor bending motions, a linear relationship was observed between the bending angle and *V*_OC_, indicating the PVA H‐TENG can serve as an angle sensor. Further investigation indicated the bending rate was also proportional to *V*_OC_; thus the device has the potential to measure both location and velocity. Figure 9b illustrates the fabrication process of an egg white‐based H‐TENG by 3D printing and injection.^[^ ^76^ ^]^ The device was integrated into clothes for wearable applications. When the TENG was subjected to pressure from 1 to 80.6 kPa, the output voltage increases linearly from 0.05 to 0.52 V, indicating it has potential for pressure sensing. Others have shown the capability of H‐TENGs to act as pressure sensors as well.^[^ ^98^, ^101^, ^130^ ^]^

H‐TENGs are also promising in health monitoring. For instance, Kim et al.^[^ ^89^ ^]^ have developed a sustainable catechol‐chitosan‐diatom hydrogel based on ocean biomaterials. Since people with Parkinson disease suffer from hand and leg shaking, M‐shape TENGs based on the developed hydrogels have been designed as self‐powered tremor sensors to monitor Parkinson disease. As shown in Figure 9c, the power spectral density of voltage signal was almost zero when the motion sensor was attached on the wrist in the normal state. With minor tremor, two peaks at low frequency were detected. Following that, several intensive peaks were further detected in severe tremor state. These data indicate that the health situation of patients can be well monitored by the H‐TENG serving as a tremor sensor.

Different from the common pressure or angle sensors, Sheng et al.^[^ ^131^ ^]^ have developed a training band sensor based on sodium alginate/zinc sulfate/poly acrylic‐acrylamide (SA‐Zn) H‐TENGs. As shown in Figure 9d, the self‐powered smart belt system consisted of signal acquisition and processing systems and real‐time display. In this regard, when the TENG was stretched, the *V*_OC_‐time curve appeared on the screen together with the applied force and calories. Consequently, a linear relationship between the output voltage and force/calorie was observed, revealing the potential of SA‐Zn TENG as a smart arm training band sensor for real‐time monitoring.

### 7.2 Electronics Powering/Charging

Unlike sensing that can be achieved by many materials, (for example, hydrogel itself as a strain/capacitance/temperature sensor^[^ ^64^ ^]^), powering/charging of portable electronics is one of the most unique and important applications of TENGs. The electricity generated by H‐TENGs harvested from human motions can also power and charge wearable electronic products, with the involvement of simple power management unit.

The typical multifunctional circuit with a rectifier and capacitor for powering and charging is schematically shown in Figure 9e. First, the circuit can be connected to the external loads such as resistors to study the DC output of TENGs at different resistances. Second, the circuit can be directly connected to LEDs to instantly light them up. Third, the circuit can be connected to a capacitor to store the generated electricity from the TENG side and switch to the other side for powering/charging. Under continuous hand tapping, the organohydrogel‐based TENG proposed by Sun et al.^[^ ^100^ ^]^ could light up tens of blue LEDs simultaneously at a subzero temperature and drive portable electronics such as timers, calculators, and thermometers with the assistance of capacitors for energy storage. In fact, almost all the H‐TENGs developed so far use similar power management circuits for electronic applications.

As an example, Li et al.^[^ ^132^ ^]^ fabricated a transparent and flexible H_3_PO_4_/PVA H‐TENG (Figure 9f) that had 26 LEDs connected in series (aligned into the “UNSW” logo) could be powered by hand tapping. The developed H‐TENG has also been used to charge a 22 µF capacitor, where 6.6 V was achieved within 6 min by simple tapping. The stored electricity could be further used to power a commercial temperature/humidity meter. Liang et al.^[^ ^130^ ^]^ have proposed a PAA/NaCl H‐TENGs as wearable smart skins for human motion energy harvesting as shown in Figure 9g. Interestingly, tens of LEDs in series could be lit by the device in both the original and stretched states, indicating the stretching did not negatively affect the device output. After storing enough electricity to a 47 µF capacitor, the smart skins could further charge an electronic watch.

Besides the single‐electrode mode, two‐electrode H‐TENGs are commonly used to power or charge electronics.^[^ ^28^, ^61^, ^74^, ^77^, ^96^ ^]^ For instance, a two‐electrode H‐TENG was composed of thermoplastic polyurethane and PDMS as the triboelectric layers, PAM hydrogel as the electrode, and PET as the supporter.^[^ ^96^ ^]^ As shown in Figure 9h, the designed device with PDMS as the interface spacer showed high transparency. Unlike other commonly reported studies, this work has used the H‐TENG to charge a capacitor with a super high capacitance of 1000 µF. Although it took more than 2000 s to charge the capacitor to 2 V, the stored energy was sufficient to power a pedometer for more than 10 min as shown by the real‐time voltage curve. Similarly, the 1000 µF capacitor with stored electricity can continuously power a digital watch for several minutes. It is noted that the sharp voltage drop in the voltage–time curve was related to initiation of the digital watch.

### 7.3 Human‐Machine Interactions

The emergence of Internet of Things and smart wearable electronics in recent years has spurred interests in human–machine interactions (HMIs).^[^ ^133^, ^134^, ^135^, ^136^ ^]^ In comparison with traditional rigid electronic devices, flexible and wearable HMIs are the future. Since hydrogels show the advantages of flexibility and stretchability, and TENGs are unique power sources, it is highly promising to integrate H‐TENGs into HMI systems.

**Figure 10:**
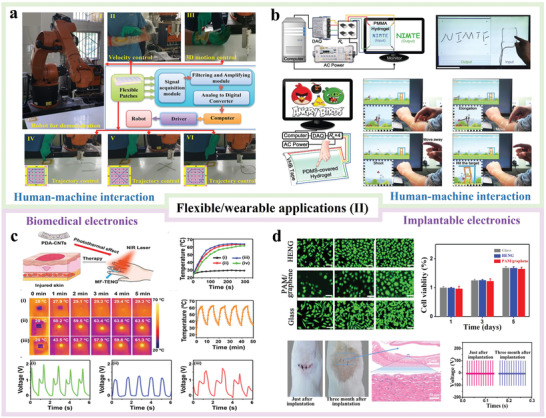
Flexible and wearable applications (II) of H‐TENGs: a) schematic diagram, flowchart and system structure, and photograph of the robotic control demonstration. Reproduced with permission.^[^ ^137^ ^]^ Copyright 2021, American Chemical Society. b) Schematic diagram of an integrated hydrogel touch screen and its applications in writing and playing Angry Birds game. Reproduced with permission.^[^ ^88^ ^]^ Copyright 2020, Willey‐VCH. c) Photothermal treatment curves and stability of hydrogels and corresponding TENGs, Reproduced with permission.^[^ ^84^ ^]^ Copyright 2021, American Chemical Society. d) Biocompatibility of PAM/graphene H‐TENG and implantation in rats. Reproduced with permission.^[^ ^83^ ^]^ Copyright 2021, Elsevier.

Chen et al.^[^ ^137^ ^]^ have proposed a self‐powered triboelectric sensing patch consisting of the starch‐based hydrogel, PDMS, and silicone rubber. This innovative patch, either in 1D (“line” shape) or 2D (“plane” shape), could detect trajectories, velocities, and displacements. Therefore, detection and control of the 3D signal can be realized by combining 1D and 2D patches, which can be further applied to robotic control and human–machine interactions. In order to verify this concept, a robotic control system was developed and is shown in **Figure** **10**a, in which the triboelectric sensing patch was connected to the signal acquisition system, computer, drive system, and robot. It was observed that the velocity control and 3D motion control could be accomplished and moreover, the trajectory tracking patch can control the robotic manipulator, so that the three different letters (C”, “X”, and “Q”) were written on a whiteboard.

Another example concerning the HMI system can be found in Gao et al.’s study,^[^ ^88^ ^]^ where a bioinspired hydrogel‐based touch pad is presented. To make a touch screen, the PDMS‐covered hydrogel touch pad was adhered to a PMMA plate on an LCD monitor. Figure 10b shows the diagram of the integrated touch screen together with the detailed connection among components including the computer, DAQ, *R*_e_, AC power and monitor. When human fingers were used to write the letters “NIMTE” on the touch screen, the high‐resolution letters appear on the monitor. More interestingly, the touch pad can be worn on the person's arm and integrated with a computer to play computer games like “Angry Bird”. As soon as the finger touched any position of the touch pad, the slingshot in the game was activated.

Similar design of self‐powered HMI systems based on the H‐TENGs has been reported by Zhang et al.^[^ ^138^ ^]^ In this work, a smart glove with five H‐TENG sensors was attached to the dorsum of the five fingers. It was obvious that the output voltage and signal of each sensor change with different gestures resulted in different squeeze deformations of H‐TENG sensors. Thus, the smart glove connected to a control board could successfully control the movement of an intelligent car by different sign language gestures.

### 7.4 Biomedical/Implantable Electronics

Owing to the unique merits of biocompatibility, biodegradability, and environmental friendliness, hydrogels are often used in biomedical and implantable applications.^[^ ^139^, ^140^, ^141^ ^]^ The advent of flexible TENGs makes it possible to be applied in similar applications, such as in vivo heartbeat monitoring,^[^ ^142^ ^]^ drug delivery,^[^ ^143^, ^144^ ^]^ tissue regeneration,^[^ ^51^ ^]^ and muscle stimulation.^[^ ^145^ ^]^ Therefore, a few researchers have also considered H‐TENGs into biomedical and implantable applications.

For example, Yang et al.^[^ ^84^ ^]^ have prepared H‐TENGs for photothermal treatment. As shown in Figure 10c, the PDA‐CNTs in the hydrogel matrix served as the photothermal conversion agents for the temperature increase of its H‐TENG. Upon irradiation with NIR laser, the temperature increased to accelerate microcirculatory blood flow and relieve pain at the injured area, which was defined as photothermal treatment. Compared to the temperature change of the pure PVA hydrogel from 20 to 29.3 °C, the temperature of the PDA‐CNTs/PVA hydrogel and TENGs significantly increased to above 60 °C under NIR irradiation. To further demonstrate the photothermal effect, the H‐TENG was attached to the finger to detect bending. It was observed that two voltage peaks were detected when the finger was in the healthy state but one peak appears if the finger was injured. After the finger undergoes photothermal treatment with the H‐TENG, the double‐peak waveform was detected again, indicating the H‐TENG could assist in the recovery of finger injury.

Alternatively, an implantable H‐TENG has been reported for battery‐free wireless nerve simulators.^[^ ^83^ ^]^ The TENG consisted of the PAM/graphene hydrogel as the electrode and PDMS as the packaging and triboelectric materials. To ensure its safety as an implantable device, the biocompatibility and biosafety of the developed TENG was investigated. As shown in Figure 10d, fibroblasts (L929) were cultured on the device, hydrogel, and glass for 1, 3, and 5 days. For easy to observe under fluorescent microscopy, the live and dead cells were stained with live/dead assays (green/red, respectively). It was found that the density, morphology, and viability of cells on the device and hydrogel were similar to those on the glass plate. In this case, the device was implanted in Sprague Dawley rats for 3 months. The output voltage of the device after implantation was almost the same as that after 3 months, thereby corroborating the excellent biocompatibility and stability in vivo. Furthermore, the implanted TENG could serve as a wireless neurostimulator without attached rectifiers, which could be further be used to inhibit pro‐inflammatory cytokines.

## 8 Challenges and Prospects

**Figure 11:**
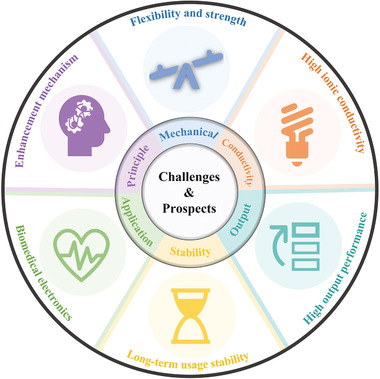
Challenges and prospects of H‐TENGs.

Based on the reported H‐TENGs so far, a comprehensive summary and comparison concerning all key parameters (such as hydrogel, dielectric material, output, transparency, stretchability, mechanical strength, conductivity, and long‐term stability) has been provided in Table S1, Supporting Information. Similar with other types of TENGs and energy harvesters, H‐TENGs also face many problems and hurdles; more work is needed to bring them to commercial fruition. **Figure** **11** simply highlights the main challenges and prospects of H‐TENGs, where the detailed discussion concerning the principles, mechanical viability, conductivity, performance, stability, and applications are summarized below.

### 8.1 Principle

With regard to the synthesis of hydrogels, both the initiators and cross‐linkers are essential components to convert the liquid precursors to solid hydrogels. This makes the components in hydrogels quite diverse, thus complicating the investigation of the internal mechanisms. In addition, a lot of studies have added many elements to enhance the mechanical and electrical properties of hydrogels. Although most of them have reported and hypothesized the impact of non‐covalent versus covalent bonds, only a few have furnished solid evidence about the existence of these bonds. Therefore, there is still not sufficient knowledge to elucidate the enhancement mechanisms unequivocally with the inclusion of these functionalizations in hydrogels in TENGs. More work is needed to better understand the resulting chemical structure and related issues pertaining to hydrogels.

### 8.2 Mechanical Properties

There is no doubt that hydrogels are highly flexible, stretchable, and shape adaptable, which bodes well for wearable applications. However, it can not be ignored that the mechanical strength of hydrogels is lower than that of many other flexible electrodes in TENGs, which limits some areas of applications. Even though double networks can be created in hydrogels by adding one or more polymers, there is still room to improve the mechanical strength of the materials. There have been attempts to add metal nanowires or 2D carbon‐based materials, but the flexibility and stretchability of the modified hydrogel can be compromised. In fact, it is difficult to balance the stretchability (>1000% of strain) and mechanical strength (>500 kPa of stress) of hydrogels. Further investigations should put more efforts on the improvement of the comprehensive mechanical properties and take inspiration from other fields that are focused on these types of advancements such as tissue engineering or aerospace material development.

### 8.3 Conductivity

As mentioned, the conductivity of hydrogels is lower than that of common flexible metal electrodes, especially organohydrogels prepared by the solvent replacement technique. Although the involvement of conductive additives (such as salts, carbon‐based, and metal‐based materials) increases the conductivity by 2–4 orders of magnitude, the conductivity of most so‐called “highly conductive hydrogels” is still only 10^−2^–10^−3^ S cm^−1^. To satisfy the requirements of high outputs and electronic applications, the expected value should be >10^−1^ S cm^−1^. Other reported strategies such as immersing hydrogels into salt solutions or building stable charge channels in hydrogels still need to be refined. Therefore, future studies should address these problems by rational formulations and designs taking into account the molecular interactions.

### 8.4 Output

At this time, the output of H‐TENGs is often lower than that of other flexible TENGs. Methods such as surface modification and tribo‐counterpart optimization can enhance the properties, but most devices are still limited by the utilization of a single‐electrode working mode. In comparison with the two‐electrode mode, the single‐electrode mode is more convenient from the perspective of device fabrication and application, albeit they exhibit lower structural figure‐of‐merit. Two‐electrode H‐TENGs may deliver better performance with the same triboelectric materials (compared to single‐electrode as H‐TENGs), but such configuration makes it impossible to use human skin—the largest positive material reported so far, which restrict integration in wearable and biomedical applications.

### 8.5 Stability

Even though the limited stability caused by drying of hydrogel has been essentially overcome, there are still two potential stability problems concerning the applications of H‐TENGs: i) physically, the low mechanical strength of hydrogels limits the long‐term stability in weight bearing or gravity applications. As heavy loads are applied continuously, H‐TENGs have a high chance to be fractured; and ii) chemically, the lack of polymer–polymer interaction in hydrogels limits the mechanical strength in comparison with bulk polymers; thus affecting the conductivity and long‐term output stability. Therefore, constructing a robust network in hydrogels is still challenging for H‐TENGs.

### 8.6 Application

By taking advantage of human motion energy harvesting, most reported H‐TENGs can be used in wearable applications such as self‐powered sensing, electronics powering, and human–machine interactions. Although only a few devices have been considered in biomedical or implantable applications for health treatment, there is still a long way to go before commercialization of biomedical H‐TENGs. Similar with other TENGs, both electrodes and triboelectric materials in H‐TENGs must meet requirements for biocompatibility and biodegradability prior to commercial application acceptance. It may be easier to satisfy these demands with hydrogels, but it is harder to match the triboelectric materials to build bio‐TENGs with high outputs. In implantable applications, factors associated with biosafety and body rejection must be considered as well.

## 9 Summary

Development of hydrogels as current collectors in TENGs has been rapid recently and there has been good progress with respect to the ionic conductivity, stretchability, flexibility, and biocompatibility. Although recent advances (such as environmental tolerance and self‐healing) are closing the gap between research and commercial applications, development of commercial hydrogels and H‐TENGs still faces challenges because of the unclear chemical nature of the interfaces, relatively poor conductivity and stability, and limited flexible/wearable applications. Nevertheless, owing to the favorable biocompatibility and environmental friendliness, hydrogels are expected to attract as much attention as other common electrodes for flexible TENGs in the future, especially biomedical and implantable applications. It is not impossible that the conductivity and output of H‐TENGs could surpass those of other flexible TENGs. However, parameters such as performance and environment stability, will be important to realize commercial applications of H‐TENGs. Simple fabrication of highly conductive and durable hydrogels will make H‐TENGs excellent alternatives for future wearable and biomedical applications.

## Conflict of Interest

The authors declare no conflict of interest.

## Supporting information

Supporting Information
