# Supplementary material for: Hydrogels as Soft Ionic Conductors in Flexible and Wearable Triboelectric Nanogenerators
Source: Adv Sci (Weinh). 2022 Feb 20;9(11):2106008. doi: 10.1002/advs.202106008 (PMC9009134; doi:10.1002/advs.202106008)
Supplement: Supplementary file 1 — Supporting Information [file ADVS-9-2106008-s001.pdf]

ADVANCED SCIENCE
Open Access

Supporting Information

for *Adv. Sci.*, DOI 10.1002/advs.202106008

Hydrogels as Soft Ionic Conductors in Flexible and Wearable Triboelectric Nanogenerators

Yinghong Wu, Yang Luo, Tyler J. Cuthbert, Alexander V. Shokurov, Paul K. Chu, Shien-Ping Feng* and Carlo Menon**

Hydrogels as Soft Ionic Conductors in Flexible and Wearable Triboelectric Nanogenerators

Yinghong Wu, Yang Luo, Tyler J. Cuthbert, Alexander V. Shokurov, Paul K. Chu, Shien-Ping Feng*, and Carlo Menon**

(Declaration of interest: none)

Dr. Y. Wu, Dr. T. J. Cuthbert, Dr. A. V. Shokurov, Prof. C. Menon

Biomedical and Mobile Health Technology Lab, Department of Health Sciences and Technology, ETH Zurich, Zurich, Switzerland. E-mail: carlo.menon@hest.ethz.ch

Dr. Y. Luo and Prof. P. K. Chu

Department of Physics, Department of Materials Science and Engineering, and Department of Biomedical Engineering, City University of Hong Kong, Hong Kong, China.

E-mail: yluo24-c@my.cityu.edu.hk

Prof. S. P. Feng

Department of Mechanical Engineering, The University of Hong Kong, Hong Kong, China.

E-mail: hpfeng@hku.hk

Department of Advanced Design and Systems Engineering, City University of Hong Kong, Kowloon, Hong Kong, China

Table S1. Comprehensive comparison of reported H-TENGs.

Ref.	Hydrogel/ionogel	Tribo-layer	E-size (cm)	Voc (V)	Jsc (mA/m^2)	Power density	Transparency (%)	Stretchability (%)	Stress (kPa)	Conductivity (S/cm)	Storage time	Storage condition	Hydrogel weight	Device output
[27]	PVA	PDMS-Al	8×8	200	3.52	2 mW	Y	35	/	/	/	/	/	/
[28]	PAM/LiCl	PDMS-Nylon	3×4	145	1.25	35 mW/m^2	(96.2-91.1)	- 19/63	(446.2/152.4)	/	1 month	30 °C/30% RH	92-89.8% (5-30 d)	99.80%
[77]	PAM/alginate	PDMS-skin	2×1	70	2.30	135 mW/m^2	(90)	(792)	(110-230)	1.25*10^{-5}	21 days	30 °C/26% RH	14.5%, (3 d)	98%
[59]	PAM/LiCl	PDMS-Al	4×4	277	13.75	4.22 W/m^2	99.6	(330)	(~370)	/	7 days	RT/desiccator	23% (1 d)	83.50%
[130]	PAA/NaCl	Ecoflex/ZnS-skin	3×5	180	43.33	6.25 W/m^2	96.9	1100	~230 (~360)	/	/	70 °C/oven	20.8% (4 h)	/
[78]	PVA/PEI	PVA/PEI-skin	2×2	70	30.20	2.79 W/m^2	90 (87)	318	32.89 MPa	5.78-6.33*10^{-9}	5 days	23.8 °C/23% RH	/	85.70%
[90]	PVA/borax	PDMS-skin	4×2.5	~20	0.24	5 mW/m^2	(88.6)	(>900)	~265	1.3*10^{-3}	14 days	ambient condition	/	100%
[92]	PVA/PDAP/MWCNT	SR-skin	3×3	95	1.11	750 mW/m^2	N	~430	~80	/	10 months	/	/	/
[60]	Chitosan/AgNWs/Cu	PDMS-skin	2×2	218	34.44	1.2/2 W/m^2	~75-90	~75-110	~180-220	4.8-2.5*10^{-3}	7 days	22°C/58% RH	8% (1 d)	88%
[105]	PAA/PDMAPS	PDMS-Al	2.5×2.5	117	22.88	1.3 W/m^2	(>90)	~800 (>400)	(726)	1.1*10^{-3}	1 month	30 °C/30% RH	100% (2 d)	99.90%
[54]	PVA/Ag NWs	Ecoflex-water/Al	5×5	78	/	0.79 mW	Y	580	338	/	/	/	/	/
[71]	BC/ZnO	Teflon-BC/ZnO	/	57.6	5.78 µA	42 mW/m^2	N	N	/	/	/	/	/	/
[61]	PVA/Na-alginate	PDMS-Al	2×5	98.6	7.30	0.98 W/m^2	~100 (>90)	~1200 (>250)	/	~3-15*10^{-3}	28 days	air condition	/	85.64%
[86]	PSGP	PSGP-PDMS	2×2	12	0.50	/	N	~900	~70		/	/	/	
[74]	PAM	PDMS-PBS/PBMA	2.5×5	~280	2.72	1.1 W/m^2	(~58)	~800	~70	/	15 days	/	/	/
[76]	egg white/NaOH	Ecoflex-skin	5×5	~20	/	/	99.8	Y	/	0.204	1 month	/	/	
[104]	PBA/PEGDA/LiTFSI	VHB-Nylon	3×3	90	1.39	55.9 mW/m^2	(91.5)	(~1036)	~170 (~330)	~10^{-6}-10^{-4}	5 months	RT	100% (100°C/5h)	99%
[97]	PAM/HEC/LiCl	SR-skin	3×3	285	17.22	626 mW/m^2	Y	1483-864	/	2.6*10^{-5}	20 days	RT	53-52% (12-20 d)	89.28%
[43]	Cellulose/PVA	VHB-latex glove	1×1.5	41	3.33	/	80	747	37.3	4.6*10^{-3}	1 month	25 °C/30% RH	60-50% (3-30 d)	100%
[96]	PAM/NaCl	PDMS-TPU	/	311.5	32.4 µA	2.7 W/m^2	(~56)	/	/	/	/	/	/	/
[49]	PAM/PDA	PTFE-Nitrile	2.5×2.5	230	19.20	4.35 W/m^2	~90	~6000	~250-300	7*10^{-3}		/	/	/
[81]	PAM/LiCl	Ecoflex-PA6	1.5×1.5	270	48.90	25 W/m^2	N	(400)	/	/	/	/	/	/
[91]	PNA/LiCl	PMA-PTFE	/	-36	0.7 µA	88 mW/m^2	N	900	2.27 MPa	6.9*10^{-3}	60 days	RT/40% RH	67% (60 d)	/
[80]	PAM/gelatin/PEDOT:PSS	SR-PU	2×3	383.8	44.83	1.25 W/m^2	>50	2850 (300)	298					/
[79]	P(MEA-co-IBA)	VHB-Latex	4×1	4	1.00	/	Y	>2000	~100-900	~10^{-4}-10^{-5}		25 °C/90% RH	100% (228 h)	/
[100]	PAM/MMT/CNT	SR-PU	4×3	86.4	0.92	41.2 mW/m^2	N	4196 (500)	~180	10^{-7}~10^{-6}	30 days	25 °C/50% RH	72~99% (30 d)	~95% (2 m)
[103]	P(MEA-co-IBA)/LiTFSI	VHB-Latex	4×1	3	0.75	/	90	1640	86-944	10^{-3}~10^{-6}		25 °C/60-90% RH	~100% (5 d)	/
[75]	hyaluronic acid (HA)	PTFE-HA	2.5×3	20	0.53	5.6 mW/m^2	/	/	/	/	9 days	RT/<15% RH	91~87% (9 d)	15-85% (6 d)
[98]	Cellulose/NaCl	VHB-glove	3×3	187	0.57	/	94	235	5.2 Mpa	4.03*10^{-2}	7 days	25 °C/59% RH	/	100%
[87]	PVA/PDAP/Graphene	PDMS/CNT -Cu	2.5×2.5	132	/	/	N	~500	85	~1-3*10^{-6}	/	/	/	/
[42]	PAM/cyclodextrin	PTFE-Latex	2.5×2.5	95	16.00	635 mW/m^2	Y	> 2000	~500-600	/	/	/	/	/
[62]	PAM/Clay	PDMS/IU-Skin	4×4	157	10.00	710 mW/m^2	Y	3360	250	/	/	/	/	/
[117]	Agarose/KCl	PTFE-chitosan/glycerol	~10 cm^2	80	2.70	0.146 mW	Y	/	/	/	/	/	/	/
[89]	CCD silica	PDMS-Al	3×3	110	4.20	29.8 mW/m^2	N	~150-170	~1-8	~1-2*10^{-3}	/	/	/	/
[131]	PAA/SA/Zn_2SO_4	Ecoflex-PMMA	4×1	30	0.125-1.25	32mW/m^2	>95	>10000	~100-150	3.4*10^{-3}		RT		100% (20 d)
[101]	Cellulose/NaCl	VHB-Latex	2×2	120	2.37	315 mW/m^2	97	220	6.8 Mpa	7.2-10.8*10^{-3}	14 days	25 °C/42% RH	85-92%, 2 weeks	100% (7 d)
[63]	PAM/PVA/NaCl	Ecoflex-skin	2×2	220-350	5.5-9.2	1.02-1.81 W/m^2	87 (75)	2333 (763)	50-120	5.93-1.02*10^{-5}	4 months	25 °C/30% RH	100%	100%
[84]	PVA/PDA-CNTs/borax	SR-skin	3×3	38.57	8.87	/	N	450	0.44	(7.65 MΩ)	/	/	/	/
[64]	PAA/Nanochitin	Polyamide-Al	3×2	71	13	1.06 W/m^2	~80-90	780	/	1*10^{-2}	/	/	/	/
[85]	PVA/MXene	Ecoflex-Kapton	2×5	230	0.27	0.33 W/m^2	N	(200)			/	/	/	/
[132]	PVA/H_3PO_4	PDMS-skin	2×2	992	112	26 W/m^2	(~95)	1058	~1.7 Mpa	1.39*10^{-2}	6 months	25-18 °C/60-40% RH	100%	/
[138]	PAM/SA/NaCl	SR-skin	/	89.7	0.53-1.58 µA	~86.1 µW	Y	1300	~75-150	1.29-19.86*10^{-3}	/	/	/	/
[82]	PAM/PVA/LiCl	SR-skin	2×2	127	2.95	302 mW/m^2	>90	~2600	~140	1.1*10^{-4}	/	/	/	/
